# The clusterin connectome: Emerging players in chondrocyte biology and putative exploratory biomarkers of osteoarthritis

**DOI:** 10.3389/fimmu.2023.1103097

**Published:** 2023-03-15

**Authors:** Patrik Kovács, Peter Natesan Pushparaj, Roland Takács, Ali Mobasheri, Csaba Matta

**Affiliations:** ^1^ Department of Anatomy, Histology and Embryology, Faculty of Medicine, University of Debrecen, Debrecen, Hungary; ^2^ Center of Excellence in Genomic Medicine Research (CEGMR), Department of Medical Laboratory Technology, Faculty of Applied Medical Sciences, King Abdulaziz University, Jeddah, Saudi Arabia; ^3^ Center for Transdisciplinary Research, Department of Pharmacology, Saveetha Dental College and Hospitals, Saveetha Institute of Medical and Technical Sciences, Chennai, India; ^4^ FibroHealth Interdisciplinary Research Programme, Fibrobesity Cluster, Research Unit of Health Sciences and Technology, Faculty of Medicine, University of Oulu, Oulu, Finland; ^5^ Department of Regenerative Medicine, State Research Institute Centre for Innovative Medicine, Vilnius, Lithuania; ^6^ Department of Joint Surgery, First Affiliated Hospital of Sun Yat-sen University, Guangzhou, Guangdong, China; ^7^ World Health Organization Collaborating Center for Public Health Aspects of Musculoskeletal Health and Aging, Université de Liège, Liège, Belgium

**Keywords:** clusterin, osteoarthritis, ingenuity pathway analysis, STRING, Cytoscape, connectome

## Abstract

**Introduction:**

Clusterin is a moonlighting protein that has many functions. It is a multifunctional holdase chaperone glycoprotein that is present intracellularly and extracellularly in almost all bodily fluids. Clusterin is involved in lipid transport, cell differentiation, regulation of apoptosis, and clearance of cellular debris, and plays a protective role in ensuring cellular survival. However, the possible involvement of clusterin in arthritic disease remains unclear. Given the significant potential of clusterin as a biomarker of osteoarthritis (OA), a more detailed analysis of its complex network in an inflammatory environment, specifically in the context of OA, is required. Based on the molecular network of clusterin, this study aimed to identify interacting partners that could be developed into biomarker panels for OA.

**Methods:**

The STRING database and Cytoscape were used to map and visualize the clusterin connectome. The Qiagen Ingenuity Pathway Analysis (IPA) software was used to analyze and study clusterin associated signaling networks in OA. We also analyzed transcription factors known to modulate clusterin expression, which may be altered in OA.

**Results:**

The top hits in the clusterin network were intracellular chaperones, aggregate-forming proteins, apoptosis regulators and complement proteins. Using a text-mining approach in Cytoscape, we identified additional interacting partners, including serum proteins, apolipoproteins, and heat shock proteins.

**Discussion:**

Based on known interactions with proteins, we predicted potential novel components of the clusterin connectome in OA, including selenoprotein R, semaphorins, and meprins, which may be important for designing new prognostic or diagnostic biomarker panels.

## 1 Introduction

Epidemiological studies have estimated that over 500 million people worldwide suffer from osteoarthritis (OA) (1). However, the true global burden of the disease is likely to be much higher (2). OA is a common inflammatory joint disorder that causes degeneration of articular cartilage and affects joint movement, resulting in significant disability (3). Despite its considerable personal, economic, and societal tolls, OA has generally been neglected. The development of therapies for OA has not made significant progress, unlike for many other chronic non-communicable diseases. Currently, there are no effective pharmacological treatments or disease-modifying OA drugs (DMOADs) available (2). The current therapeutic approaches include exercise, weight loss, and education. OA patients are generally administered non-steroidal anti-inflammatory drugs (NSAIDs) to reduce inflammation and alleviate joint pain. The lack of effective therapies likely stems from the heterogeneous nature of the disease and incomplete understanding of its pathophysiology (4). This can be facilitated by identifying clinical, biological, or medical markers specific to disease phenotypes. Soluble biomarkers are of interest and many candidate biomarkers have been identified. Systemic biomarkers have the potential to report the overall burden of disease and therefore provide holistic endpoints for generalized disease analyses (5). One such systemic biomarker that has recently attracted special attention in the context of OA is *clusterin* (6).

Clusterin (also known as apolipoprotein J and several other aliases) is a multifunctional holdase chaperone glycoprotein present in almost every bodily fluid, interstitial fluid, and intracellularly (7, 8). It is the first extracellular chaperone to facilitate the clearance of misfolded extracellular proteins, similar to heat shock proteins (HSP) inside the cell (9). Molecular chaperones are characterized by their selective binding to non-native protein conformations to form stable complexes, thus inhibiting irreversible aggregation (10). The importance of extracellular chaperones is underpinned by the fact that more than 40 human degenerative diseases are associated with the deposition of fibrillar proteinaceous aggregates called amyloids, including Alzheimer’s disease (AD) and Parkinson’s disease (PD) (10). In addition to the brain and central nervous system, amyloids also affect many tissues and organs, including the musculoskeletal system (11). Amyloid deposits derived from transthyretin (TTR) and apolipoprotein A-1 (APOA1) are frequently found in knee joints of patients with OA (12). The molecular structure of clusterin comprises molten globular-like features with putative amphipathic α-helices, which allow it to interact with the hydrophobic regions of proteins exposed to stress (13, 14). Upon binding, clusterin either stabilizes these proteins or facilitates their degradation (15). Clusterin has traditionally been associated with neuroprotection, mainly because of its role in clearing misfolded proteins, such as β-amyloid in AD (16). In addition, clusterin levels are associated with myocardial infarction (17). Clusterin is also associated with pain and inflammation. For example, lower clusterin serum concentrations were linked to higher pain scores in patients with erosive hand OA (18). Clusterin is used as a translational preclinical biomarker of various conditions, such as renal injury (19, 20), AD (21), cognitive disorders (22), and inflammatory conditions, such as vasculitis (23). Although clusterin levels in body fluids clearly reflect pathophysiological processes in many settings, and its use as a biomarker or biomarker candidate seems promising, it is unsuitable as a single unique diagnostic tool.

Clusterin is a moonlighting protein with many functions, including lipid transport, cell differentiation, regulation of apoptosis, and clearance of cellular debris, and seems to play a protective role in ensuring cellular survival (7). However, the possible involvement of clusterin in arthritic and rheumatic diseases has been relatively understudied, and only two published studies have examined its potential as a biomarker for cartilage lesions (24, 25). Therefore, further research is needed to study the roles of the secreted and intracellular forms of clusterin in osteoarticular tissues and to confirm whether clusterin could be used as a biomarker candidate in OA. Clusterin has been reported to be secreted by articular cartilage and chondrocytes (26, 27). Exposure to the pro-inflammatory cytokine interleukin-1β (IL-1β) resulted in reduced levels of clusterin precursor, but increased levels of mature clusterin (~35 kDa) released into the secretome of equine articular cartilage explants (26). Using *in vitro* models of low-grade inflammation in OA (which relies on a combination of tumor necrosis factor-α (TNF-α) and IL-1β), clusterin secretion into the secretome was attenuated (27). Clusterin is a robust marker of local synovial inflammation, as its level is significantly elevated in synovial fluid samples from patients with OA (28).

Despite accumulating (and often seemingly controversial) data, clusterin may have cytoprotective and anti-apoptotic effects, or other moonlighting functions that have not been studied in OA (6). Given the significant potential of synovial and systemic clusterin as biomarkers of OA, a more detailed analysis of its complex network in an inflammatory environment, specifically in the context of OA, is required. In order to address this, in the present study, we first used the STRING database and Cytoscape (29) to map and visualise the clusterin connectome. QIAGEN Ingenuity Pathway Analysis (IPA; Qiagen, Germantown, MD, USA) software, an advanced bioinformatics tool with a massive built-in scientific literature-based knowledge database, was employed to analyze and study clusterin-associated signalling networks in OA. The purpose of this study was to identify, based on the connectome and interactome available in public databases and the IPA knowledgebase, putative novel entities that could be developed into biomarkers (or rather panels of biomarkers) in OA. To this end, the interactions between clusterin and its partners in the broader connectome and interactome networks were investigated, highlighting their putative or established roles in arthritic diseases.

## 2 Methods

### 2.1 Elaborating the clusterin connectome using STRING and Cytoscape

We first employed the STRING database (version 11.5; string-db.org) to search for known protein interactions and Gene Ontology (GO) annotations of clusterin. STRING (Search Tool for the Retrieval of Interacting Genes/Proteins) is a biological database and web resource of known and predicted protein–protein interactions, and it is not exclusive to joint tissues or OA. We then used the PubMed query service in Cytoscape to import the top 50 protein interaction data for clusterin (confidence cut-off:0.4; network type: full-string network; query: clusterin; or clusterin AND osteoarthritis) based on publications indexed in PubMed. Owing to spatial limitations, the interactants identified using the PubMed query are included and discussed in the context of inflammatory joint disorders in the **Supplementary Material**.

### 2.2 Ingenuity pathway analysis

Clusterin interactome-associated genes were decoded using the Ingenuity Pathway Analysis (IPA) knowledge database (Qiagen, USA). The core analysis module was selected to identify significant upstream and downstream effects of the clusterin interactome on canonical pathways, diseases, biofunctions, causal networks, unique non-directional networks, tox functions, and pathological functions (30, 31). Fisher’s exact test with a *p*-value cut-off ≤ 0.05 and Benjamini-Hochberg (B-H) correction were used to calculate statistical significance. Activation or inhibition of canonical signaling pathways, diseases and disorders, molecular and cellular functions, and physiological system development and function were computed based on the Z-score algorithm of IPA and compared with an idealized activation or inhibition pattern for a signaling pathway, disease/disorder, or biological function. The IPA molecular activity predictor tool (MAP) (31) was used to assess the effects of clusterin activation or inhibition on the signaling pathways associated with OA.

### 2.3 Transcription factor analysis

Genome-wide RNA sequencing datasets of normal and OA-affected joint articular cartilage were downloaded from the Gene Expression Omnibus (GSE114007^1^) (32). Normalized read counts of individual samples (18 normal and 20 OA) were averaged before the analysis. The GeneHancer (GH) regulatory elements were then evaluated. Clusterin promoter/enhancer GH08J027610 had the highest gene association score of 255.90. The GeneHancer dataset contains 248 potential transcription factors that can bind to this sequence of genes of interest. The expression levels of these factors were compared between the control and OA groups.

## 3 Results and discussion

### 3.1 Clusterin has multiple interacting partners and is involved in diverse biological processes

**Figure 1:**
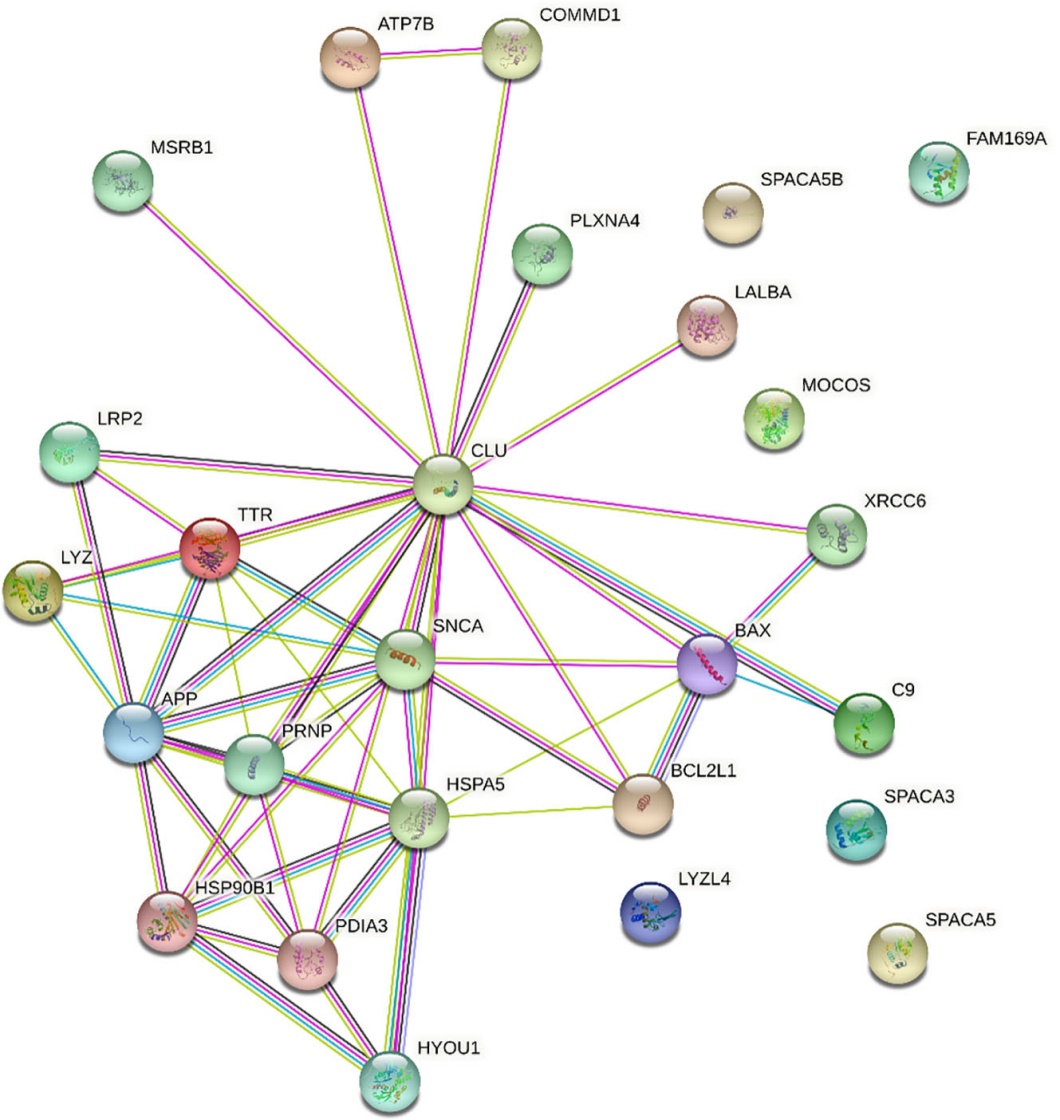
STRING interaction network for clusterin. Only direct connections are discussed. APP,amyloid-beta A4 protein; ATP7B, ATPase copper transporting beta; BAX, BCL2 associated X apoptosis regulator; BCL2L1, BCL2 like 1; C9, complement protein C9; CLU, clusterin; COMMD1, copper metabolism Murr1 domain; FAM169A, family with sequence similarity 169 member A; HSP90B1, heat shock protein 90 beta family member 1; HSPA5, heat shock protein family A (Hsp70) member 5; HYOU1, hypoxia up-regulated 1; LALBA, α-lactalbumin; LRP2, low-density lipoprotein receptor related protein 2; LYZ, lysozyme; LYZL4, lysozyme like 4; MOCOS, molybdenum cofactor sulfurase; MSRB1, methionine sulfoxide reductase B1; PDIA3, protein disulfide isomerase family A member 3; PLXNA4, plexin A4; PRNP, prion protein; SNCA, synuclein alpha; SPACA3, sperm acrosome associated 3; SPACA5, sperm acrosome associated 5; SPACA5B, sperm acrosome associated 5B; TTR, transthyretin; XRCC6, X-Ray Repair Cross-Complementing Protein 6. Edge colours are as follows: known interactions: light blue, from curated databases; magenta, experimentally determined; predicted interactions: green, gene neighbourhood; red, gene fusions; dark blue, gene co-occurrence; others: lime, text-mining; black, co-expression; purple, protein homology.

Clusterin has 455 known interacting partners according to the STRING database; however, we only processed the top 25 interactants in this study^2^. The clusterin connectome, based on the top 25 interactants, contained 53 edges (connections), and the average node (protein) degree was 4.08, with a PPI enrichment *p*-value < 1.0e-16 (**Tables 1**, **2**; **Figure 1**). The top hits in the clusterin network included intracellular chaperones (HSPA5 and HSP90B1) and aggregate-forming proteins (APP, SNCA, and PRNP), which is not surprising given their historic association with neurodegenerative disorders. Below we are focusing on the direct connections of clusterin.

**Table 1:** Network statistics of the STRING connectome network of clusterin.

number of nodes (proteins):	26
number of edges (connections):	53
average node degree:	4.08
avg. local clustering coefficient:	0.646
expected number of edges:	8
PPI enrichment p-value:	< 1.0e-16

**Table 2:** Entities in the STRING connectome network of clusterin ranked by node degree.

Entity	Protein name	Node degree
*CLU*	clusterin	19
*HSPA5*	heat shock protein family A (Hsp70) member 5	10
*APP*	amyloid-beta A4 protein	9
*SNCA*	synuclein alpha	9
*HSP90B1*	heat shock protein 90 beta family member 1	7
*PRNP*	prion protein	7
*TTR*	transthyretin	7
*BAX*	BCL2 associated X apoptosis regulator	6
*PDIA3*	protein disulfide isomerase family A member 3	6
*BCL2L1*	BCL2 like 1	4
*HYOU1*	hypoxia up-regulated 1	4
*LYZ*	lysozyme	4
*LRP2*	LDL receptor related protein 2; melagin	3
*ATP7B*	ATPase copper transporting beta	2
*C9*	complement protein C9	2
*COMMD1*	copper metabolism Murr1 domain	2
*XRCC6*	X-ray repair cross-complementing protein 6	2
*LALBA*	α-lactalbumin	1
*MSRB1*	methionine sulfoxide reductase B1	1
*PLXNA4*	plexin A4	1
FAM169A	family with sequence similarity 169 member A	0
LYZL4	lysozyme like 4	0
MOCOS	molybdenum cofactor sulfurase	0
SPACA3	sperm acrosome associated 3	0
SPACA5	sperm acrosome associated 5	0
SPACA5B	sperm acrosome associated 5B	0

Direct connections are highlighted in *italics*.

#### 3.1.1 Heat shock proteins and intracellular chaperones

Among known interacting partners, heat shock protein family A (Hsp70) member 5 (HSPA5, also known as 78 kDa glucose-regulated protein, GRP78; BiP) has the highest number of edges (10). HSPA5 is a chaperone in the endoplasmic reticulum (ER) lumen, which is known to regulate clusterin stability under ER stress (33) and is involved in the molecular mechanisms of ER stress induced during chondrogenesis (34). HSP90B1 (Grp94) also has a very high number of edges (7). HSP90B1 is involved in ER stress triggered by excessive mechanical load and hypoxia in chondrocytes (35).

#### 3.1.2 Amyloidogenic proteins and protein misfolding

Amyloid-β A4 protein (APP) is one of the top entities in the clusterin connectome (36). The clusterin–amyloid β-peptide complex interacts with low-density lipoprotein (LDL) receptor-related protein 2 (LRP2; megalin, known to act as a clusterin receptor), which offers a mechanism to clear the pathological accumulation of aggregates (37). Autoantibodies against LRP2 have been detected in 87% of patients with rheumatoid arthritis (RA) and 15% of patients with OA, indicating that these anti-LRP2 autoantibodies may play pathological roles by inhibiting the protein reabsorbing function of LRP2 in the proximal tubule (38). α-synuclein (SNCA) also forms a major component of amyloid plaques in AD and PD, and this process is blocked by clusterin (39). Clusterin interacts with extracellular α-synuclein fibrils and limits their uptake by astrocytes (40). Transthyretin (TTR) is an amyloidogenic protein. Clusterin is known to interact with aggregated forms of TTR, and serum clusterin levels in patients with transthyretin amyloid cardiomyopathy are significantly lower than those in healthy controls (41). TTR deposition in articular cartilage has been reported to increase disease severity in a murine model of OA (42). Moreover, both clusterin and TTR levels were higher in synovial fluid samples of patients with knee OA than in those with hand OA, indicating that they are involved in similar molecular pathways during OA pathogenesis (43). Clusterin interacts with amyloidogenic variants of lysozyme (LYZ) (44). Lysozyme has long been known to be present in the cartilage ECM (45), and cartilage degradation leads to increased serum and synovial fluid lysozyme levels in patients with OA (46). Clusterin is also involved in the folding/unfolding pathway of the extracellular protein α-lactalbumin (LALBA) (47).

Protein disulfide isomerase family A member 3 (PDIA3, ERp57), an oxidoreductase involved in native disulfide bond formation, is required for efficient clusterin oxidative folding (48). In the case of protein misfolding in the ER of chondrocytes, ECM proteins aggregate, resulting in ER stress, and the unfolded protein response (UPR) is initiated. Persistent ER stress is a pathogenic mechanism underlying OA (49). Hypoxia upregulated 1 (HYOU1), a marker of protein misfolding under cellular stress, is involved in the chondrocyte response to IL-1α (50).

#### 3.1.3 Anti-apoptotic proteins

Clusterin has a well-known anti-apoptotic role, partly because it reduces the activity of the pro-apoptotic protein BCL2 associated X apoptosis regulator (BAX) (51, 52). However, the regulatory mechanisms of BAX underlying chondrocyte apoptosis in OA remain largely unknown (53), and clusterin involvement in this pathway has not been implicated in OA chondrocytes. In contrast, using a text-mining-based approach, anti-apoptotic BCL2L1 (BCL-XL) was recently identified as a gene that could be exploited as a potential drug target in OA (54) and is known to regulate apoptosis through the BCL-XL protein in kidney cells (55).

#### 3.1.4 Copper homeostasis

ATPase copper-transporting β (ATP7B) is an important regulator of intracellular Cu homeostasis (15). COMMD1 (Copper metabolism Murr1 domain 1) is expressed in most tissues and plays a role in controlling protein degradation and stability (56). Clusterin and COMMD1 interact with ATP7B independently. As a consequence of these interactions, degradation of misfolded Cu-ATPase molecules is facilitated, which is an important factor in the quality control of ATP7B required for the maintenance of normal copper homeostasis (15). Literature on the function of copper transporters in chondrocytes is sparse (57), although a genetic predisposition to physiologically higher circulating copper and zinc status may increase the risk of OA (58). Significantly higher Cu concentrations have been detected in the synovial fluid of patients with OA than in healthy subjects (59). Therefore, Cu levels and transporter status in combination with clusterin should be further investigated in the context of OA. In contrast, COMMD1 is an important mediator of NF-κB signalling, a key player in inflammatory pathways, and clusterin has been linked to COMMD1 protein levels (60). Clusterin also has a complex regulatory interaction with NF-κB signalling (61).

#### 3.1.5 Complement system

The complement system is involved in host defense mechanisms that aim to eliminate potentially harmful structures from the body. Clusterin potently inhibits terminal complement assembly by blocking complement protein (C9), thereby reducing the rate of complement-mediated cytolysis and providing higher levels of protection (62). Complement protein C9 has been described in the hypertrophic zone of the epiphyseal growth plate (63). C9 appears to be predominantly present in SC5b-9 complexes in synovial membrane samples from patients with OA (64). In cases of acute arthritis, such as OA flare-up, marked C9 deposits were detected in the synovium; however, C9 deposits were not found in chronic conditions associated with degenerative diseases, such as OA (65).

#### 3.1.6 DNA repair

Clusterin is also implicated in DNA repair. Given the often fatal consequences of DNA breaks, several pathways exist for the recognition and repair of these lesions. One such pathway involves the DNA-dependent protein kinase (DNA-PK) complex, which consists of a catalytic subunit and heterodimeric Ku autoantigen comprising Ku70 (XRCC6) and Ku80 proteins (66). Clusterin was identified as an interacting partner of Ku70, likely initiating complex signalling mechanisms leading to cell death (66). In colon cancer, interleukin 6 (IL-6) affects pro-survival pathways by modulating the expression and molecular interactions between the pro-apoptotic factor BAX, DNA repair proteins Ku70/86, and clusterin (67). However, no experimental data are available on the role of clusterin in mediating repair pathways involving Ku70 in OA.

#### 3.1.7 Selenium homeostasis

Selenoprotein R (SelR, also known as methionine sulfoxide reductase B1, MSRB1) plays an important role in maintaining intracellular redox balance by reducing the R-form of methionine sulfoxide. Given that selenium is an essential trace element, selenoproteins that mediate its metabolism are involved in key cellular functions such as redox homeostasis (68). SelR interacts with clusterin (69). Co-overexpression of SelR and clusterin in an AD model significantly decreased intracellular ROS levels. Furthermore, the interaction between clusterin and β-amyloid peptide was confirmed, suggesting a putative effect of SelR and β-amyloid peptide *via* clusterin (69). Appropriate selenium levels are required to maintain cartilage development and homeostasis (68), and experimental evidence suggests that selenoproteins are expressed in chondrocyte cell lines *in vitro* (70). Selenium deficiency is linked to the development of Kashin–Beck disease (KBD), which is an endemic osteoarthropathy (prevalent in low-selenium areas of China, North Korea, and Siberia in Russia) caused by disturbances in the closure of the epiphyseal plate and manifests as skeletal deformities and movement disorders (71). Certain polymorphisms in selenoprotein genes are associated with a higher risk of KBD (72). Furthermore, a cross-sectional analysis of dietary selenium intake revealed that high dietary selenium consumption may be associated with an increased risk of OA (73). However, SelR has not yet been directly discussed in the context of OA development.

#### 3.1.8 Semaphorins

Plexins are receptors of the semaphorin family of signalling proteins (74). Plexin A4 (PLXNA4) acts as a clusterin receptor in the central nervous system and is an emerging therapeutic target for AD (68). Although plexins in chondrocytes have only been partially mapped, semaphorin-3A (Sema3A) has been implicated in OA chondrocyte physiology because excessive Sema3A signalling stimulated by the pro-inflammatory cytokines interleukin-1β (IL-1β) and tumor necrosis factor-α (TNF-α) promotes apoptosis (75). Sema4D has recently been shown to be involved in chondrocyte apoptosis triggered by lipopolysaccharide (LPS) (76). Therefore, elucidating aberrant semaphorin signalling in the context of plexins and clusterin may lead to the identification of new targets in OA.

### 3.2 A text-mining approach further expanded the clusterin connectome

**Figure 2:**
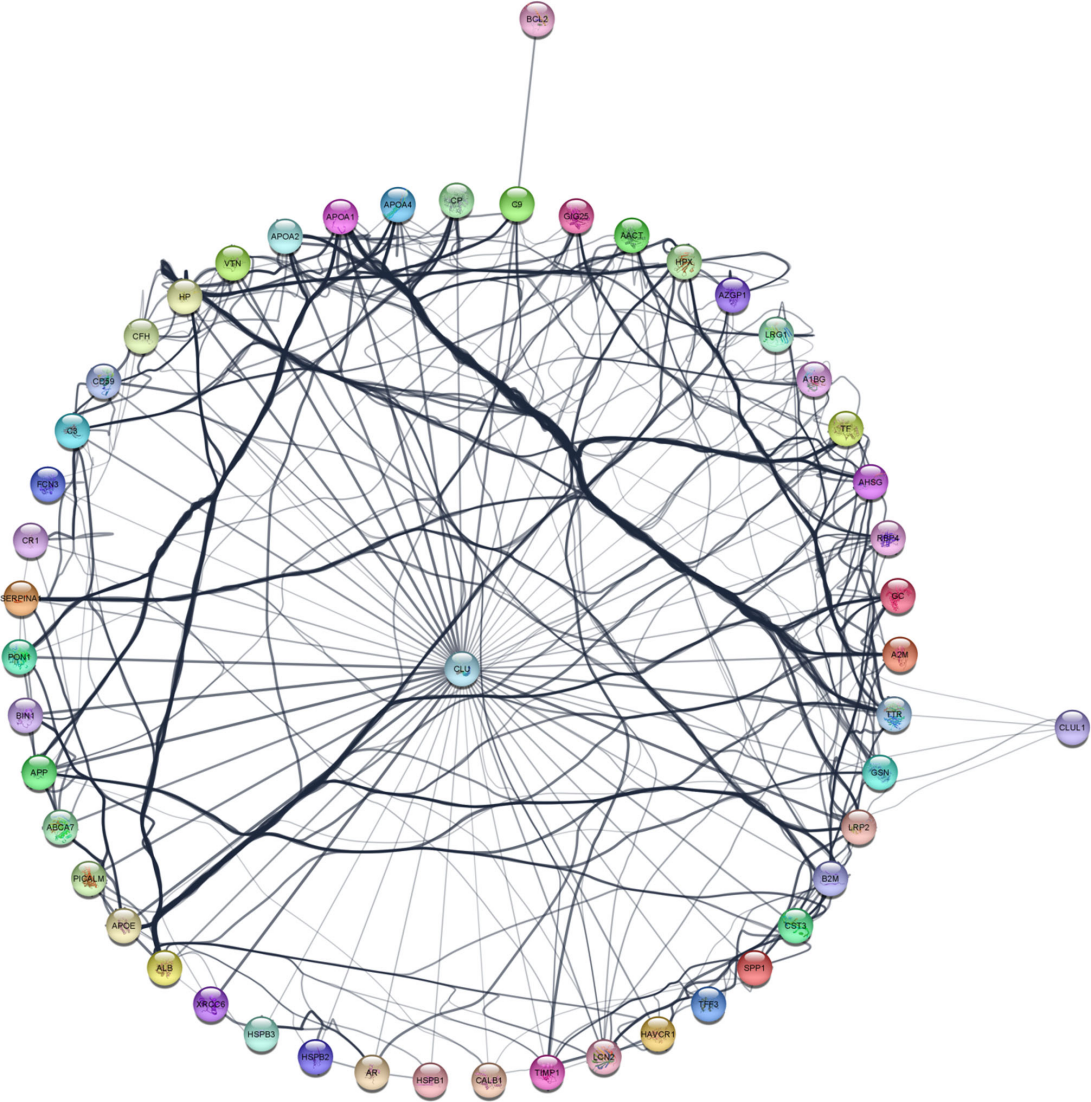
The expanded clusterin connectome retrieved using the PubMed query service in Cytoscape. See further details in text.

To further identify interacting partners with clusterin, we used the PubMed query text-mining service in Cytoscape to expand the clusterin connectome (**Figure 2**). In the expanded connectome, additional interacting partners or proteins that were discussed together with clusterin in the research articles indexed in PubMed were retrieved (**Table 3**). Among these, APP, TTR, LRP2, C9, and XRCC6 have been discussed above. Owing to spatial limitations, most of the interacting partners identified using this text-mining approach are discussed in detail in the **Supplementary Material**.

**Table 3:** Entities in the extended connectome network of clusterin ranked by node degree as identified by text-mining using Cytoscape.

Entity	Protein name	Node degree
**CLU**	**clusterin**	47
ALB	serum albumin	42
APOE	apolipoprotein E	34
HP	haptoglobin	33
APOA1	apolipoprotein A-I	32
SERPINA1	serpin peptidase inhibitor, clade A; alpha-1-antitrypsin	31
APOA4	apolipoprotein A-IV	30
C3	complement C3	30
A2M	alpha-2-macroglobulin	29
**TTR**	**transthyretin**	28
CP	ceruloplasmin	28
HPX	hemopexin	28
GC	vitamin D-binding protein-macrophage activating factor	26
AHSG	alpha-2-HS-glycoprotein	26
CST3	cystatin C	26
VTN	vitronectin	26
RBP4	retinol binding protein 4	25
APOA2	apolipoprotein A-II	25
GSN	gelsolin	25
TF	beta-1 metal-binding globulin/transferrin	25
SERPINA3	serpin peptidase inhibitor, clade A, member 3	24
**APP**	**amyloid beta (A4) precursor protein**	24
B2M	beta-2-microglobulin	23
A1BG	alpha-1-B glycoprotein	21
AZGP1	alpha-2-glycoprotein 1, zinc-binding	21
LRG1	leucine rich alpha-2-glycoprotein 1	21
**C9**	**complement C9**	21
**LRP2**	**low density lipoprotein receptor-related protein 2**	18
LCN2	lipocalin 2	16
CFH	complement factor H	14
TIMP1	tissue inhibitor of metalloproteinases 1	13
SPP1	secreted phosphoprotein 1	13
PON1	paraoxonase 1	12
BIN1	box-dependent myc-interacting protein 1	10
HAVCR1	hepatitis A virus cellular receptor 1	10
CD59	CD59 molecule	9
AR	androgen receptor	8
TFF3	trefoil factor 3	7
CR1	complement component (3b/4b) receptor 1	6
ABCA7	ATP-binding cassette, sub-family A (ABC1), member 7	6
PICALM	phosphatidylinositol binding clathrin assembly protein	6
CLUL1	clusterin like 1	5
FCN3	ficolin 3	5
CALB1	calbindin 1	5
**XRCC6**	**X-ray repair cross-complementing protein 6**	4
HSPB2	heat shock 27kDa protein 2	4
HSPB3	heat shock 27kDa protein 3	4
HSPB1	heat shock 27kDa protein 1	3
BCL2	Bcl-2	1

Entities that have been identified in **Table 2** (CLU, APP, TTR, LRP2, C9, and XRCC6) are highlighted in **bold**.

### 3.3 Gene ontology annotations for the clusterin connectome reveal key biological pathways and molecular functions

GO annotations for human clusterin were retrieved from QuickGo Web Services^3^. The top biological processes included “positive/negative regulation of protein-containing complex assembly,” “positive regulation of gene expression,” “positive regulation of receptor-mediated endocytosis,” “protein targeting to lysosome involved in chaperone-mediated autophagy,” “negative regulation of cell death,” “positive/negative regulation of amyloid fibril formation,” “negative regulation of response to endoplasmic reticulum stress,” “positive regulation of proteasomal ubiquitin-dependent protein catabolic process,” “protein stabilization,” and chaperone-mediated protein folding” (**Table 4A**). Clusterin is involved in the following key molecular pathways: “protein binding,” “signaling receptor binding,” “amyloid-beta binding,” “protein carrier chaperone,” “protein-containing complex binding,” “tau protein binding,” “low-density lipoprotein particle receptor binding,” “chaperone binding,” and “misfolded protein binding” (**Table 4B**).

**Table 4:** Biological pathway (A) and molecular pathway (B) GO terms associated with clusterin.

GO term	GO name
A. Biological pathways	
GO:0005515	protein binding
GO:0005102	signaling receptor binding
GO:0001540	amyloid-beta binding
GO:0140597	protein carrier chaperone
GO:0044877	protein-containing complex binding
GO:0048156	tau protein binding
GO:0050750	low-density lipoprotein particle receptor binding
GO:0051087	chaperone binding
GO:0051787	misfolded protein binding
GO:0031625	ubiquitin protein ligase binding
GO:0051082	unfolded protein binding
GO:0046982	protein heterodimerization activity
B. Molecular pathways	
GO:0031334	positive regulation of protein-containing complex assembly
GO:0010628	positive regulation of gene expression
GO:0048260	positive regulation of receptor-mediated endocytosis
GO:0061740	protein targeting to lysosome involved in chaperone-mediated autophagy
GO:0060548	negative regulation of cell death
GO:0090201	negative regulation of release of cytochrome c from mitochondria
GO:1905908	positive regulation of amyloid fibril formation
GO:1905907	negative regulation of amyloid fibril formation
GO:1905895	negative regulation of cellular response to tunicamycin
GO:1905892	negative regulation of cellular response to thapsigargin
GO:1903573	negative regulation of response to endoplasmic reticulum stress
GO:1901216	positive regulation of neuron death
GO:0032436	positive regulation of proteasomal ubiquitin-dependent protein catabolic process
GO:0042981	regulation of apoptotic process
GO:0002434	immune complex clearance
GO:0051092	positive regulation of NF-kappaB transcription factor activity
GO:0050821	protein stabilization
GO:0061077	chaperone-mediated protein folding
GO:0043065	positive regulation of apoptotic process
GO:2000060	positive regulation of ubiquitin-dependent protein catabolic process
GO:0042127	regulation of cell population proliferation
GO:0002376	immune system process
GO:0006958	complement activation, classical pathway
GO:0006915	apoptotic process
GO:0045087	innate immune response
GO:0006956	complement activation
GO:0006629	lipid metabolic process
GO:1902004	positive regulation of amyloid-beta formation
GO:0001836	release of cytochrome c from mitochondria
GO:0031333	negative regulation of protein-containing complex assembly
GO:0051788	response to misfolded protein
GO:0043691	reverse cholesterol transport
GO:1902230	negative regulation of intrinsic apoptotic signaling pathway in response to DNA damage
GO:0001774	microglial cell activation
GO:0000902	cell morphogenesis
GO:0045429	positive regulation of nitric oxide biosynthetic process
GO:1902998	positive regulation of neurofibrillary tangle assembly
GO:0017038	protein import
GO:0032760	positive regulation of tumor necrosis factor production
GO:0032286	central nervous system myelin maintenance
GO:0051131	chaperone-mediated protein complex assembly
GO:0061518	microglial cell proliferation
GO:1901214	regulation of neuron death
GO:1900221	regulation of amyloid-beta clearance
GO:1902949	positive regulation of tau-protein kinase activity
GO:1902847	regulation of neuronal signal transduction
GO:1902430	negative regulation of amyloid-beta formation
GO:0009615	response to virus
GO:2001244	positive regulation of intrinsic apoptotic signaling pathway
GO:0097193	intrinsic apoptotic signaling pathway

### 3.4 The clusterin network in the IPA knowledgebase

#### 3.4.1 Overall clusterin network in the IPA knowledgebase

We then analyzed the overall clusterin interactome in the IPA knowledge base and identified additional pathways and interacting partners. In the overall molecular network of clusterin, the top canonical pathways were “colorectal cancer metastasis signaling,” “regulation of the epithelial mesenchymal transition by growth factors pathway,” “pancreatic adenocarcinoma signaling,” “IL-12 signaling and production in macrophages,” and “glucocorticoid receptor signaling” (**Table 5A**). These results are in line with the well-established role of clusterin in tumor biology.

**Table 5:** Top canonical pathways of clusterin in the overall network (A), in connective tissue development and function (B), and in inflammation of joints (C).

A. Overall Network		
**Name**	***p-*value**	**Overlap**
Colorectal cancer metastasis signaling	1.24E-27	16.3 % 40/246
Regulation of the epithelial mesenchymal transition by growth factors pathway	1.54E-27	19.1 % 36/188
Pancreatic adenocarcinoma signaling	6.70E-27	26.9 % 29/108
IL-12 Signaling and Production in Macrophages	3.98E-23	21.5 % 28/130
Glucocorticoid receptor signaling	1.16E-20	11.4 % 38/333
B. Connective Tissue Development and Function		
**Name**	***p*-value**	**Overlap**
Pancreatic adenocarcinoma signaling	9.01E-31	17.4 % 19/109
Hepatic fibrosis signaling pathway	1.04E-26	6.2 % 23/368
Regulation of the epithelial mesenchymal transition by growth factors pathway	5.16E-26	10.1 % 19/188
Colorectal cancer metastasis signaling	3.32E-25	7.9 % 20/253
Chronic myeloid leukemia signaling	4.62E-25	15.5 % 16/103
C. Inflammation of Joints		
**Name**	***p*-value**	**Overlap**
Colorectal cancer metastasis signaling	3.35E-25	8.3 % 21/253
Atherosclerosis signaling	4.41E-24	13.4 % 17/127
IL-12 Signaling and Production in Macrophages	5.71E-22	12.1 % 16/132
Regulation of the epithelial mesenchymal transition by growth factors pathway	4.44E-21	9.0 % 17/188
Glucocorticoid receptor signaling	1.27E-19	5.7 % 19/336

The top upstream regulators were IL-6, CLU, TGFB1, TP53, and EZH2 (**Table 6A**). Interleukin-6 (IL-6) has been reported to influence pro-survival pathways in colon cancer progression *via* Bax, Ku70/86, and clusterin (67). IL-6 is especially relevant in the context of OA; an increase in IL-6 serum levels has been associated with decreased physical function and increased risk of knee OA progression (77). Transforming growth factor β1 (TGFB1) regulates clusterin expression (78–80). TGFB1 is an essential factor in chondrogenesis and cartilage maintenance, and a recent study confirmed that a SNP associated with OA susceptibility affects TGFB1 expression by influencing its enhancer (81). The tumor suppressor protein p53 (TP53) represses clusterin expression, which may be important for p53-mediated cell death (82). p53 has a well-established role in OA (82). EZH2, a histone methyltransferase involved in polycomb repressor complex 2 (PRC2), represses clusterin expression; therefore, aberrant upregulation of EZH2 may contribute to the progression of various tumors (83). EZH2 is upregulated in OA (84); however, the link between EZH2, clusterin, and OA progression has not yet been established.

**Table 6:** Top upstream regulators and casual network of clusterin in the overall network (A), in connective tissue development and function (B), and in inflammation of joints (C).

A. Overall Network	
Upstream Regulators	
**Name**	***p-*value**
IL6	2.97E-31
CLU	5.14E-29
TGFB1	2.92E-28
TP53	1.21E-22
EZH2	3.52E-22
Causal Network	
**Name**	***p*-value**
PPARG	7.68E-35
UCHL1	5.73E-34
TXNIP	1.10E-33
PIAS4	7.29E-32
DICER1	1.27E-31
B. Connective Tissue Development and Function	
Upstream Regulators	
**Name**	***p*-value**
TGFB1	1.85E-37
PD98059	1.10E-36
IGF1	4.57E-34
HRAS	1.53E-32
EGF	1.53E-32
Causal Network	
**Name**	***p*-value**
ADAM12	1.41E-35
HTATIP2	1.23E-34
EPHA4	3.13E-34
zibotentan	8.13E-34
BMS-387032	9.00E-34
C. Inflammation of Joints	
Upstream Regulators	
**Name**	***p*-value**
TNF	4.56E-37
IL6	4.79E-36
curcumin	2.99E-33
APP	6.40E-33
beta-estradiol	1.00E-32
*C. Inflammation of Joints*	
Causal Network	
**Name**	***p*-value**
CD276	1.18E-35
MEP1A	1.32E-35
carteolol	5.72E-35
sesamol	2.60E-34
IL6	4.09E-34

The entities in the causal network of clusterin were PPARG, UCH1, TXNIP, PIAS4, and DICER1 (**Table 6A**). Clusterin overexpression upregulates the adipogenic marker peroxisome proliferator-activated receptor γ (PPARG) during adipocyte differentiation (85). PPARG signalling is involved in skeletal muscle regeneration *via* growth/differentiation factor 3 (GDF3) (86). PPARG expression is upregulated in synovitis, indicating its role in mediating tissue recovery (87). Although ubiquitin carboxyl-terminal hydrolase (UCL1), thioredoxin-interacting protein (TXNIP), and DICER1 are among the top members of the causal network, we did not find a direct association between these factors and clusterin. Nevertheless, TXNIP, an inhibitor of antioxidant activity, is downregulated by sirtuin 6 (SIRT6) in chondrocytes (88). Furthermore, TXNIP forms a complex with DDIT/REDD1, an endogenous inhibitor of mTOR that regulates cellular stress responses; the TXNIP/REDD1 complex is required for the activation of autophagy in chondrocytes, but its expression is reduced in OA (89). A protein inhibitor of activated STAT (PIAS4/PIASY) interferes with the binding of NF-κB, an important regulator of inflammation, to its target genes (60). As previously discussed, clusterin may mediate COMMD1, which induces NF-κB destabilization and proteasomal degradation (60). DICER-dependent pathways play critical roles in chondrocyte proliferation and differentiation during skeletal development (90).

The top five networks with the involvement of clusterin were as follows: “cell death and survival, cellular assembly and organization, cancer,” “cancer, organismal injury and abnormalities, cellular development,” “cellular development, connective tissue development and function, tissue development,” “cell death and survival, lipid metabolism, molecular transport,” and “cellular assembly and organization, DNA replication, recombination, and repair, cellular compromise” (**Table 7A**).

**Table 7:** Top networks associated with clusterin in the overall network (A), in connective tissue development and function (B), and in inflammation of joints (C).

A. Overall Network		
	Associated Network	Functions Score
1	Cell death and survival, cellular assembly and organization, cancer	41
2	Cancer, organismal injury and abnormalities, cellular development	34
3	Cellular development, connective tissue development and function, tissue development	28
4	Cell death and survival, lipid metabolism, molecular transport	26
5	Cellular assembly and organization, DNA replication, recombination, and repair, cellular compromise	26
B. Connective Tissue Development and Function		
	**Associated Network**	**Functions Score**
1	Cellular development, cellular growth and proliferation, connective tissue development and function	37
2	Cellular development, cellular growth and proliferation, lymphoid tissue structure and development	19
3	Cell-to-cell signaling and interaction, carbohydrate metabolism, cellular development	12
4	Cellular development, connective tissue development and function, skeletal and muscular system development and function	10
5	Cellular development, cellular growth and proliferation, cancer	10
C. Inflammation of Joints		
	**Associated Network**	**Functions Score**
1	Connective tissue disorders, inflammatory disease, inflammatory response	48
2	Connective tissue disorders, organismal injury and abnormalities, skeletal and muscular disorders	31
3	Connective tissue disorders, inflammatory disease, inflammatory response	21
4	Cellular growth and proliferation, cancer, organismal injury and abnormalities	21
5	Cellular movement, skeletal and muscular system development and function, cellular development	4

#### 3.4.2 Connective tissue development and function

Given that one of the top networks above was “cellular development, connective tissue development and function, and tissue development,” we repeated the IPA knowledgebase analysis focusing on *connective tissue development and function*. In this analysis, the top five canonical pathways were as follows: “pancreatic adenocarcinoma signaling,” “hepatic fibrosis signaling pathway,” “regulation of the epithelial mesenchymal transition by growth factors pathway,” “colorectal cancer metastasis signaling,” and “chronic myeloid leukemia signaling” (**Table 5B**).

The top upstream regulators were TGFB1, PD98059, IGF-1, HRAS, and EGF (**Table 6B**). TGFB1, an upstream regulator of clusterin, has been discussed previously. PD98059, an inhibitor of mitogen-activated protein kinase kinase (MEK1/MAPKK), abrogated clusterin-stimulated proliferation, indicating that clusterin may activate the extracellular signal-regulated kinase 1/2 (ERK1/2) pathway (91). As discussed previously, clusterin stimulates MMP-9 expression *via* ERK1/2 and NF-κB pathways (92). In an *in vitro* model of OA, ADAMTS and MMP upregulation correlated with the activation of ERK1/2 signalling, and PD98059 reversed the overexpression of matrix metalloproteinases (93). Insulin-like growth factor-1 (IGF-1) is also known to induce clusterin expression (94) and is involved in protecting cells from premature senescence (95). IGF-1 plays key roles in cartilage by promoting chondrocyte proliferation, enhancing ECM production, and inhibiting chondrocyte apoptosis, and is therefore highly relevant in OA therapy (96). Induction of the HRAS proto-oncogene represses clusterin expression in a MEK/ERK and methylation-dependent manner, indicating that DNA hypermethylation of the clusterin promoter is controlled by oncogenic signalling pathways (97). HRAS is involved in modulating chondrocyte apoptosis, senescence, and ECM degradation *via* MAPK signalling in OA (98). Epidermal growth factor (EGF) regulates clusterin expression *via* the Ras/ERK/AP-1 signalling pathway (99). While EGF signalling plays an important role in endochondral bone formation and joint homeostasis, conflicting results on its role in OA have been reported, which is likely attributable to the activation of specific downstream molecules as well as crosstalk with other signalling pathways (100).

The entities in the causal network were ADAM12, HTATIP2, EPHA4, Zibotentan, and BMS-387032 (**Table 6B**). We discussed the inclusion of ADAMs in an extended clusterin connectome (see **Supplementary Material**). HTATIP2 is an oxidoreductase required for tumor suppression in gliomas (101). Ephrin type-A receptor 4 (EPHA4) plays an emerging role in OA. Activation of EPHA4 signalling attenuates pro-inflammatory cytokine and MMP production in synoviocytes and augments the expression of chondrogenic genes in chondrocytes (102). Zibotentan is an endothelin A receptor antagonist (103). In vascular smooth muscle cells, endothelin was found to be significantly differentially expressed in response to clusterin (104). Endothelin-1 signalling plays an emerging role in OA pathogenesis by stimulating the expression of MMP-1 and MMP-13 (105). BMS-387032 is a potent inhibitor of cyclin-dependent kinases (CDK) 2, 7, and 9 (106). CDK inhibitors reduce the injury response after joint trauma, indicating that this pathway can be exploited for the prevention and/or treatment of early OA (107).

The top five networks in the clusterin interactome in the context of connective tissue development and function were as follows: “cellular development, cellular growth and proliferation, connective tissue development and function,” “cellular development, cellular growth and proliferation, lymphoid tissue structure and development,” “cell-to-cell signaling and interaction, carbohydrate metabolism, cellular development,” “cellular development, connective tissue development and function, skeletal and muscular system,” and “cellular development, cellular growth and proliferation, cancer” (**Table 7B**).

#### 3.4.3 Molecules regulated by clusterin in the IPA knowledgebase

**Figure 3:**
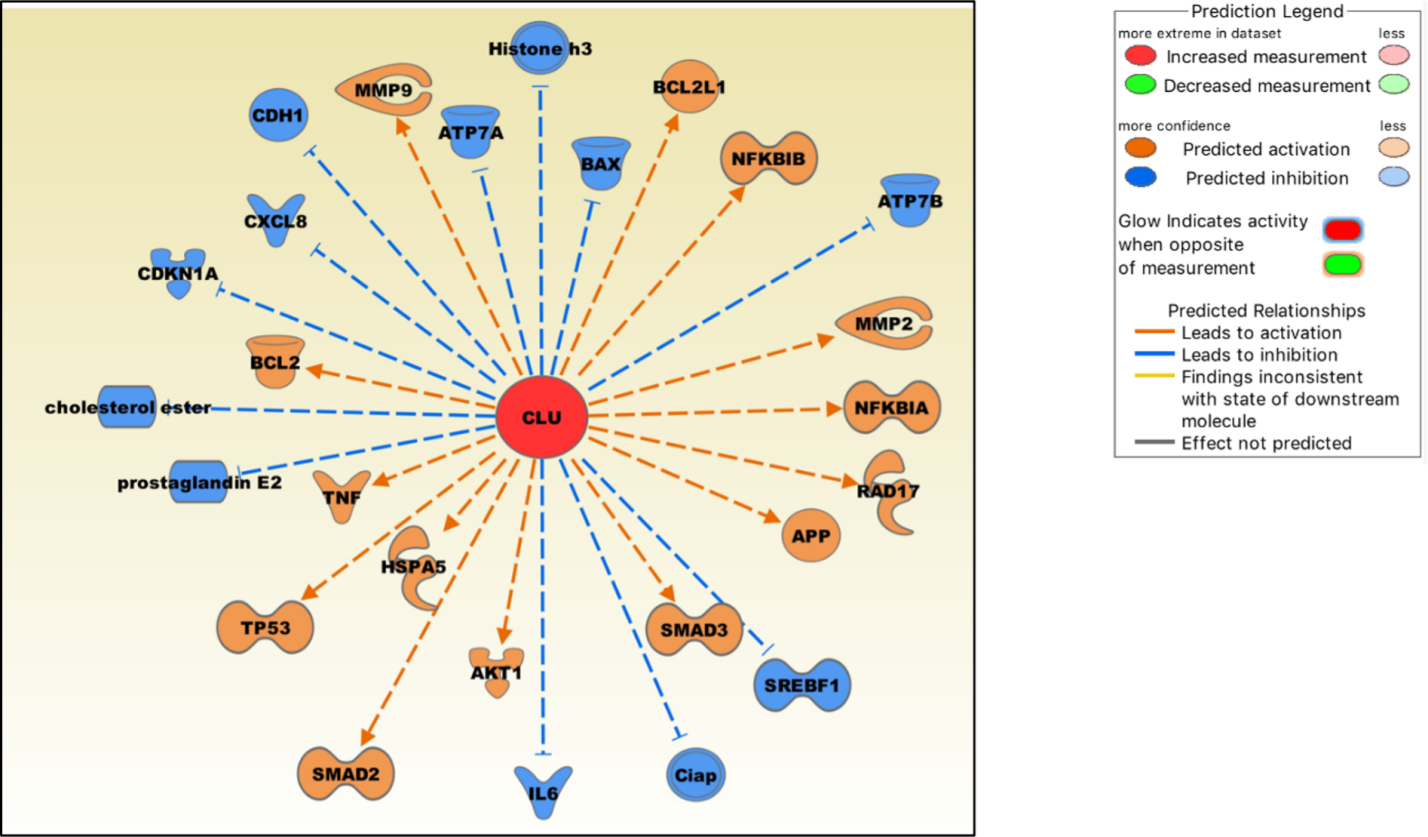
Molecules either up-regulated (orange) or down-regulated (blue) by clusterin based on the IPA knowledgebase. Image generated by the IPA software.

We extracted these molecules from the IPA knowledgebase, and their expression levels were modulated by clusterin, as shown in the published literature (**Figure 3**). In OA, clusterin-dependent regulation of several molecules is particularly relevant.

Clusterin increases the production of TNF-α and MMP-9 in macrophages (108), and both proteins are critical mediators of the OA pathophysiology (109). In cultured human fibroblast-like synoviocytes, clusterin knockdown by siRNA increased the production of pro-inflammatory cytokines IL-6 and IL-8 (CXCL8), indicating that clusterin plays a negative regulatory role in NF-κB-regulated cytokine production (24). Clusterin inhibits NF-κB signalling by stabilizing IκBs in neuroblastoma cells (110). Clusterin decreases the production of PGE_2_ (prostaglandin E_2_) (111), a principal mediator of inflammation, in RA and OA (112). Clusterin gene silencing in human OA chondrocytes shifted the cell phenotype towards hypertrophy and increased apoptosis, downregulated NF-κB-regulated genes, and increased MMP13 and TNF-α levels, suggesting a protective role of clusterin in these cells (113). Clusterin is involved in upregulating MMP-2 and downregulating E-cadherin expression in tumor cells (114). As discussed previously, clusterin and COMMD1 interact to downregulate ATP7A and ATP7B copper-transporting ATPases, thereby mediating Cu homeostasis (15). Overexpression of clusterin blocks TNF-α-mediated induction of p21 (CDKN1A) and abrogates proteolytic activation of the apoptosis regulator BAX, rendering clusterin-overexpressing breast cancer cells significantly more resistant to cytokines (115). Clusterin regulates the expression of proteins in mitochondrial apoptosis pathways, such as Bcl‐2, BAX, Bcl‐xL and caspase‐9, and phosphorylation of Akt (116). Clusterin blocks hepatic lipid accumulation by inhibiting SREBP-1c expression, suggesting that it may be a suitable target for preventing hepatic fat accumulation in insulin-resistant patients (117).

Clusterin is also an emerging modulator of TGF-β signalling that regulates SMAD2/3 proteins (118). These proteins are essential for the formation and maintenance of healthy cartilage and SMAD3 mutations are associated with OA (119). Clusterin overexpression increased SMAD2/3 protein levels *via* enhancing TGF-β-induced transcriptional activity (118). Clusterin is also involved in stabilising SMAD2/3. In tumor cells, clusterin plays a protective role against ER stress-induced apoptosis by interacting with glucose-regulated protein 78 (GRP78; also known as HSPA5), a central regulator of the unfolded protein response (120). GRP78 is upregulated in advanced OA, suggesting that chondrocytes experience ER stress during its pathogenesis (121). Clusterin may also be involved in regulating cellular cholesterol homeostasis under both normal and pathological conditions (122). Cholesterol homeostasis plays a key role in skeletal development, the dysregulation of which contributes to the development of cartilage diseases, including OA (123).

### 3.5 Clusterin network in OA (molecular activity prediction) in the IPA knowledgebase

**Figure 4:**
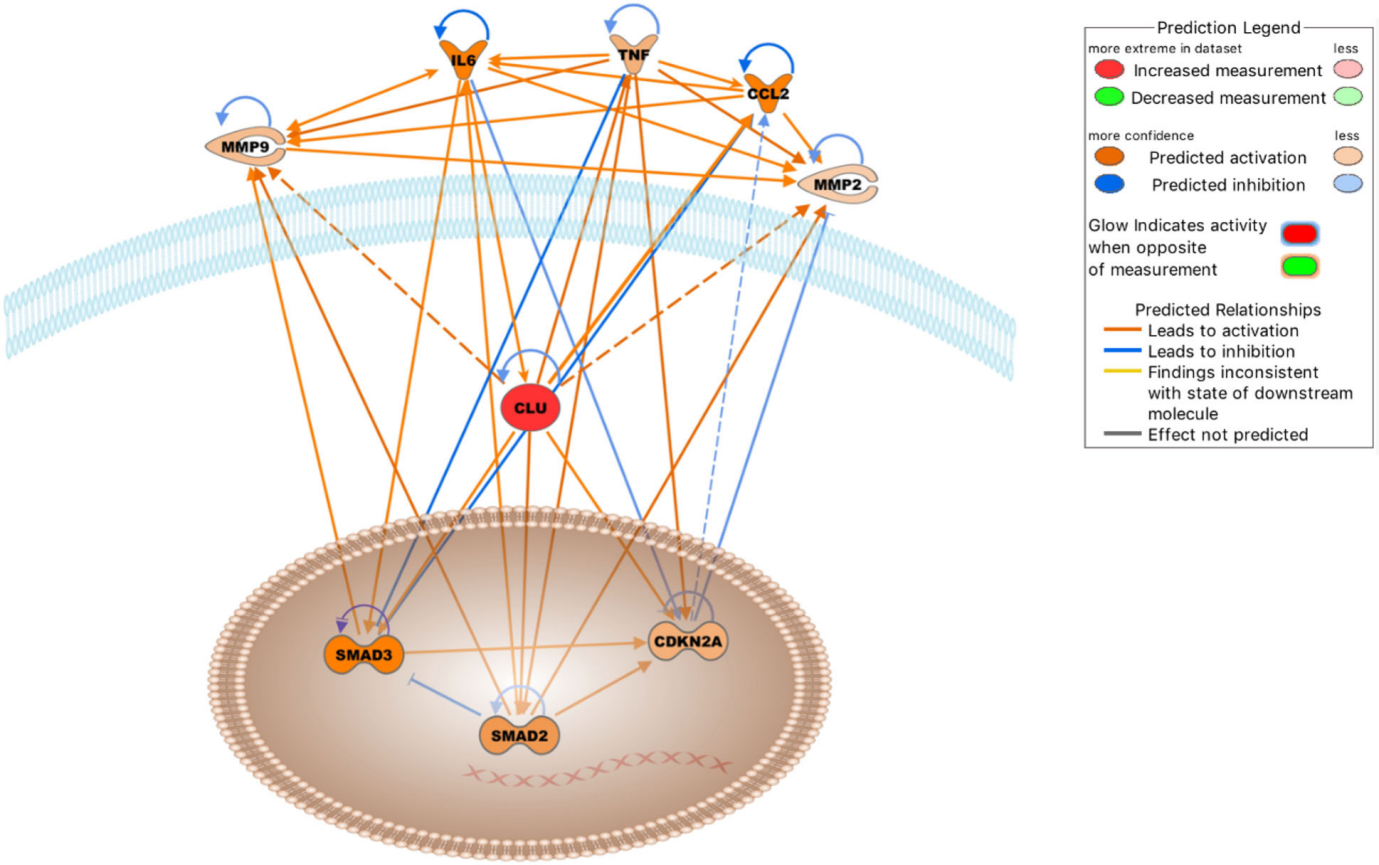
Molecules either activated (orange lines) or inhibited (blue lines) in the clusterin network in the context of OA based on the IPA knowledgebase (Dashed lines: probable activation). Image generated by the IPA software.

We also analyzed the clusterin connectome in the context of joint inflammation (**Figure 4**). In this context, the top canonical pathways, similar to the previous two analyses, included “colorectal cancer metastasis signaling,” “atherosclerosis signaling,” “IL-12 signaling and production in macrophages,” “regulation of the epithelial mesenchymal transition by growth factors pathway,” and “glucocorticoid receptor signaling” (**Table 5C**).

The top upstream regulators were TNF, IL6, curcumin, APP, and β-estradiol (**Table 6C**). TNF, IL-6, and APP have previously been discussed in the context of the clusterin connectome. Curcumin, an anti-inflammatory compound derived from *Curcuma* spp., has been used in clinical trials to determine its effectiveness in OA patients. Patients experienced improvement in pain, physical function, and quality of life after taking curcumin; these effects are attributable to the fact that curcumin blocks activation of the NF-κB system in chondrocytes (124). As discussed previously, various components of NF-κB signalling are directly associated with clusterin. β-estradiol regulates clusterin expression (125). The prevalence of OA was higher in women than in men in all age groups. In a study performed on total knee arthroplasty samples, sex differences were found in the synovial fluid levels of vitamin D metabolites, cytokines, and metalloproteinases, as well as in the cellular expression of 17β-estradiol receptors (126).

Entities in the causal network of clusterin were CD276, MEP1A, carteolol, sesamol, and IL6 (**Table 6C**). CD276 (also known as B7-H3), a member of the immunoglobulin superfamily, has been identified in chondrocytes by our group and others (127, 128). The expression of CD276 is correlated with poor prognosis in several pathologies, including RA (129), and is implicated as a promising therapeutic target for autoimmune diseases. Meprin α (MEP1A) is the largest secreted extracellular protease that hydrolyzes, activates, or inactivates several cytokines and growth factors. For example, it cleaves various MMPs, ADAMs, BMPs, DKK-1, collagen, syndecans, and fibronectin, many of which are relevant in the context of OA. It also cleaved clusterin (130). However, meprins have not been implicated in OA. Carteolol is a nonselective β-adrenoceptor antagonist. The β-adrenergic receptor signalling pathway plays a detrimental role in temporomandibular joint OA (131) and regulates cartilage catabolism induced by IL-1β (132). Sesamol, a natural organic compound present in sesame seeds and sesame oil, exerts its protective effect by blocking MMP expression *via* NF-κB or ERK/p38 MAPK signalling (133), offering a potential chondroprotective strategy in OA.

In the context of joint inflammation, the top five networks were as follows: “connective tissue disorders, inflammatory disease, inflammatory response,” “connective tissue disorders, organismal injury and abnormalities, skeletal and muscular disorders,” “connective tissue disorders, inflammatory disease, inflammatory response,” “cellular growth and proliferation, cancer, organismal injury and abnormalities,” and “cellular movement, skeletal and muscular system development and function, cellular development” (**Table 7C**). These networks further support the relevance of clusterin in inflammatory joint disease.

IPA identified five key regulatory networks that are especially relevant in the context of OA. These interactions have been previously described in detail, and are briefly discussed below.

**1. IL-6 increases clusterin expression.** Clusterin has been shown to be regulated by the pro-inflammatory cytokine IL-6 in various models (67, 134, 135). Importantly, an association between clusterin and IL-6 has been documented in cultured human fibroblast-like synoviocytes (FLSs) (24). Knockdown of clusterin using siRNA induced a significant and reproducible increase in the baseline production of IL-6 in FLSs, highlighting its negative regulatory role in NF-κB-dependent cytokine production.

**2. Clusterin induces the expression of TNF-**α **and other cytokines.** NF-κB, which is activated by extracellular stimuli including inflammatory cytokines such as TNF-α, is a key regulator of gene expression programs that culminate in stress-like responses. IKKs are upstream mediators of NF-κB activation (136). Clusterin has been identified as one of several genes that is dependent on IKK activation upon stimulation by TNF-α, suggesting that this pathway could protect against immune complex-mediated inflammatory reactions (137). TNF-α significantly alters the biogenesis of clusterin, leading to the appearance and nuclear accumulation of a 50–53 kDa uncleaved, non-glycosylated, disulfide-linked isoform (138). TNF-α also increases the level of the cytoplasmic 36–38.5 kDa clusterin isoform (139). These anomalous intracellular forms are likely attributable to aberrant glycosylation of clusterin released from the secretory system into the cytosol under ER stress (8). Conversely, exogenous clusterin increased TNF-α release from activated microglial cells (140). Clusterin binds to TNF-α in the BioPlex human interactome network (141). Furthermore, clusterin upregulates the expression of chemotactic cytokines such as monocyte chemotactic protein-1 (MCP-1) and macrophage inflammatory protein-1β (MIP-1β), regulated upon activation, normal T cell expressed and secreted (RANTES), and TNF-α in macrophages (108). However, clusterin is a negative regulator of TNF-α in OA chondrocytes, as increased TNF-α levels have been detected in clusterin-silenced human OA chondrocytes (113). Based on the above, the effects of clusterin on cytokine (*e.g*., TNF-α) production depend on the cell type, disease state, and the interplay between other intracellular pathways, depending on the available upstream or downstream factors.

**3. Clusterin modulates the enzymatic activity and expression of MMPs.** In both RA and OA, inflammatory cytokines, such as IL-1β and TNF-α stimulate the production of ECM-degrading MMPs (142). A direct interaction between clusterin and MMP-9 has been demonstrated in human epithelial cells, where clusterin binding prevents stress-induced MMP-9 aggregation and inhibits MMP-9 enzymatic activity. Clusterin also inhibits the enzymatic activities of MMP-2, MMP-3, and MMP-7. Treatment with pro-inflammatory cytokines (such as IL-1β and TNF-α) reduced clusterin expression (143). In contrast, clusterin knockdown resulted in a significant downregulation of MMP-2 in human hepatocellular carcinoma cells (114). Clusterin facilitates the nuclear translocation of NF-κB along with IκB-α degradation and phosphorylation in macrophages, leading to MMP-9 upregulation. Notably, only the intact secretory form of clusterin promotes MMP-9 activation; glycosylation-deficient and non-glycosylated recombinant clusterin is unable to stimulate MMP-9 (92, 108). Clusterin increases MMP-9 activity by increasing the phosphorylation status of p38 MAPK in platelet-stimulated colon carcinoma cells, thereby increasing invasion (144). The carcinogenic factor dinitrosopiperazine increased the binding of CLU to MMP-9 and upregulated MMP-9 expression *via* clusterin (145). In contrast, increased MMP13 levels were observed in human OA chondrocytes following clusterin silencing (113). These data also highlight that MMP regulation by clusterin is cell-type- and context-dependent, relying on concurrent active signal transduction pathways.

**4. Clusterin is required for CDKN2A up-regulation.** Forkhead box transcription factor L2 (FOXL2) stimulates clusterin expression in pituitary tumors. Clusterin induces the expression of cyclin-dependent kinase inhibitor p16 (CDKN2A), thereby inhibiting pituitary cell proliferation (146). FOXL2 is a major transcription factor in various developmental pathways, including bone and cartilage development, and its actions overlap with those of SOX9 (147). FOXL2 regulates ECM components (Col1a2, Col3a1, Col4a1, fibronectin, and laminin) in the ovaries (148). Senescence-promoting p16^INK4a^ (CDKN2A), which is expressed in synovial tissue, is an OA marker and its somatic deletion partially protects against cartilage degeneration (149).

**5. Clusterin increases the expression of SMAD2 and SMAD3.** As discussed earlier, clusterin regulates SMAD2/3 proteins, which are key modulators of cartilage formation, by interacting with TGF-β type II receptor. Clusterin can also stabilize SMAD2/3 proteins, potentially *via* proteasomal degradation (118).

### 3.6 Clusterin transcription factor analysis in OA

Ten transcription factors that can bind to promoter/enhancer GH08J027610 were selected based on our criteria, which were two major conditions: first, the expression level to be in the top 10 percentile among the average OA read counts; and second, the fold change value to be above 1.0, on average, of OA samples normalized to the average of normal ones. These transcription factors include CTBP1, KLF6, MBD2, REST, SMARCE1, SOX5, TEAD1, XRCC5, ZEB1, and ZNF280D. According to the PANTHER™ Gene Ontology classification (150), the term ‘metabolic process’ (GO:0008152) was the most abundant, with eight of the 10 genes annotated by this term. Other notable categories included ‘biological regulation’ (GO:0065007), ‘cellular process’ (GO:0009987), and ‘developmental process’ (GO:0032502), all of which included at least three of the 10 genes. Finally, according to the Signor 2.0 curated interactions database (151), GDNF is a transcriptional up-regulator of clusterin (based on data obtained in *Rattus norvegicus* (152)). According to the datasets we analyzed, GDNF did not show robust expression in any of the groups investigated (normal *versus* OA cartilage); however, it is still notable that its expression level increased from the 23^rd^ percentile in normal cartilage to the 35^th^ percentile in OA cartilage in terms of ranking within the total transcriptome.

## 4 Summary

Clusterin, a multifunctional holdase chaperone, is an enigmatic protein with a wide range of functions that exerts its moonlighting role by acting in concert with an array of interacting proteins reviewed in this article. Clusterin is a moonlighting protein because, in addition to its conventional role as an extracellular chaperone in proteostasis, it is involved in a variety of other functions, including cell survival, complement inhibition, and cell differentiation (8). Clusterin is enigmatic, because we are far from understanding the actions of its cytoplasmic form. Here, we used an *in silico* approach to examine the interaction partners and connections of clusterin in OA. Clusterin interacts with a large number of proteins, as is evident from this study, as well as the additional information contained in the **Supplementary Material** that further analyses an extended list of clusterin interactants. Clusterin interactome is likely to expand further with the identification of new partners.

Based on known interactions with proteins, we predicted potentially novel components of the clusterin connectome in OA that may be important for designing new prognostic or diagnostic biomarker panels. The cytoprotective role of clusterin during cellular stress could be attributed to several mechanisms, such as anti-apoptotic signaling *via* Bax and/or Ku70, protection against oxidative stress, inhibition of the membrane attack complex (MAC) of locally activated complement proteins, and binding to stressed/misfolded proteins in a chaperone-like manner, preventing their aggregation. As discussed previously, clusterin has anti-apoptotic activity in various models by preventing Bax from entering the mitochondria or by blocking Bax phosphorylation *via* activation of the PI3K-AKT pathway (7). At the same time, however, clusterin also has pro-apoptotic functions by binding to Ku70, promoting active cell death through a caspase 3-dependent pathway (7). One of the most important roles of clusterin is the regulation of NF-κB activity. NF-κB-induced gene expression has been widely documented to contribute to the pathogenesis of inflammatory diseases including OA (24). Given the emerging role of clusterin in the regulation of apoptosis and NF-κB signalling, it is a potentially interesting and important target for RA and OA therapy. Understanding and defining the exact role(s) of this multifunctional protein in the pathogenesis of these two arthritic diseases are crucial.

Some of the interactions discussed in this paper have been described in various *in vitro* models or *in vivo* experiments, and have not (yet) been identified in the context of OA. However, given that, in addition to the complex role of clusterin, these interacting partners are also key players in OA pathogenesis and/or prognosis, it is likely that these interactions also exist in OA-affected joints. The interacting partners that warrant further experimental confirmation of OA are as follows. Clusterin is a known interacting partner of Ku70, which is a component of the DNA-dependent protein kinase complex that triggers cell death. However, the role of clusterin in mediating repair pathways involving Ku70 in OA has not yet been investigated. Selenoprotein R (SelR) maintains intracellular redox balance in cells, and clusterin interacts with SelR. Co-overexpression of SelR and clusterin significantly decreased intracellular ROS levels. Although selenoproteins are expressed in chondrocyte cell lines, SelR itself has not been explored in the context of OA. Other interesting candidates that have emerged as potentially relevant markers are semaphorins, a versatile group of proteins involved in various processes including axonal growth and bone development. Sema3A signalling stimulated by IL-1β and TNF-α promotes apoptosis, and Sema4D has recently been shown to be involved in chondrocyte apoptosis. Elucidating aberrant semaphorin signalling in the context of clusterin may lead to the identification of new targets in OA. Furthermore, meprins, which are extracellular proteases involved in connective tissue homeostasis, cleave procollagen I, amyloid precursor protein (APP), and IL-6R (153). Despite their roles as extracellular proteases and their specific targets, meprins have not been implicated in OA, highlighting the need for further research.

## 5 Conclusions and perspectives

Given that it is unlikely that any single biomarker can be sufficiently sensitive and specific to fulfil all needs, such as early disease detection, prediction of disease progression, and monitoring response to therapy as an effective intervention marker, it is likely that a combination of biochemical and imaging markers will ultimately be used serially and in combination to optimize OA drug development and patient therapy in OA. This is probably the case with clusterin; as a single biomarker, it will likely be insufficient to aid in the diagnosis and prognosis of patients with OA.

It is now evident that clusterin levels in bodily fluids are altered in various pathological conditions. Furthermore, it is involved in a plethora of intracellular signalling pathways, the outcomes of which are context-dependent. A growing body of evidence suggests that clusterin is a promising biomarker for OA (6). The diverse roles of this protein should be carefully considered in future translational and clinical orthopaedic studies, and special attention should be paid to its involvement in other comorbidities. It is important that future biomarker studies, especially when clusterin levels are measured in bodily fluids such as serum or urine, should not correlate clusterin levels exclusively to the process of OA pathogenesis. Clusterin in the synovial fluid is likely to be more suitable for further development as a biomarker candidate.

One such comorbidity is obesity. Clusterin plasma concentration is closely associated with metabolic disorders, such as obesity, and a high-fat and high-sucrose diet (Western diet) leading to diet-induced obesity is accompanied by increased clusterin levels in mice (154). Aberrant metabolism has been linked to different phenotypes of OA, and obesity is one of the most important risk factors of the disease (155). Clusterin is increasingly used as a biomarker for obesity-related AD (156), and different levels of clusterin in the CSF are associated with various stages of AD pathology (157). Although adipocyte-derived adipokines, including clusterin, may play a direct role in OA pathology, future studies are needed to determine whether clusterin is a viable biomarker for at least certain OA phenotypes or molecular endotypes and if it offers a key link between obesity, metabolic disease, and OA.

Future research is necessary on clusterin as a soluble biomarker candidate to establish whether it can provide new insights into OA pathogenesis progression and determine whether it can be used to aid in defining molecular endotypes, along with other biomarker candidates, perhaps the network of proteins identified and discussed in this article, including selenoprotein R, semaphorins, and meprin. Thus, clusterin will be a great asset for future research on OA pathogenesis, progression and potentially also for assessing responses to therapeutic interventions.

## Data availability statement

Publicly available datasets were analyzed in this study. This data can be found here: Gene Expression Omnibus, GSE114007.

## Author contributions

Conceptualization: AM, CM. conducting the research: network/pathway analysis using STRING/Cytoscape, PK. IPA analysis, PP. transcription factor analysis, RT literature search, CM. significant contribution to discussions, all authors. All authors contributed to the article and approved the submitted version.

## Footnotes

1
https://www.ncbi.nlm.nih.gov/geo/query/acc.cgi?acc=GSE114007 (Last accessed: 19-Feb-2023)

2
https://version-11-5.string-db.org/cgi/network?networkId=bBHxWsZnQ9sG (Last accessed: 19-Feb-2023)

3
https://www.ebi.ac.uk/QuickGO/annotations?geneProductId=P10909 (last accessed: 19-Feb-2023)
