# Supplementary material for: The clusterin connectome: Emerging players in chondrocyte biology and putative exploratory biomarkers of osteoarthritis
Source: Front Immunol. 2023 Mar 15;14:1103097. doi: 10.3389/fimmu.2023.1103097 (PMC10081159; doi:10.3389/fimmu.2023.1103097)
Supplement: Supplementary file 1 [file DataSheet_1.pdf]

Supplementary Material for

The Clusterin Connectome: Emerging Players in Chondrocyte Biology and Putative Exploratory Biomarkers of Osteoarthritis

A text-mining approach to further expand the clusterin connectome

To further identify interacting partners with clusterin, we used the PubMed query text-mining service in Cytoscape to expand the clusterin connectome. In the expanded connectome, additional interacting partners or proteins that were discussed together with clusterin in research articles indexed in PubMed were retrieved (see *Table 3* in the main document). However, due to spatial limitations, most of the interacting partners identified using this text-mining approach are discussed in detail in the Supplementary material below.

Although serum albumin (ALB) has the highest number of nodes (42), this is likely attributable to the fact that clusterin levels are often monitored in various diseases in the serum where albumin is present in very high quantities. Nevertheless, albumin is a protein that co-purifies with clusterin from stressed plasma, forming clusterin-client complexes, which are proposed to act as a vehicle to clear damaged misfolded extracellular proteins (1).

Clusterin is also known as apolipoprotein J (APOJ) based on its ability to bind to lipid molecules. There is a clear association between apolipoproteins A1 and E (APOA1 and APOE), as well as clusterin with cerebral amyloid angiopathy (2). APOA1, APOE, and clusterin have also been identified as proteins bound to fibrin clots in the plasma, indicating that they act as extracellular chaperones (3). APOA1 is an amyloidogenic protein that may also form amyloid deposits in the knee joints of OA patients (4). The antioxidant activity of high-density lipoprotein (HDL) is partially attributed to the enzyme paraoxonase/arylesterase1 (PON1). PON1 is closely associated with a specific HDL subfraction which also contains APOA1 and clusterin (5). PON1 and clusterin were present in higher concentrations in synovial fluid samples from patients with knee OA than in synovial fluid from with 1st carpometacarpal joint OA (6). In a study which analysed synovial fluid samples 12 months after anterior cruciate ligament transection (ACLT), APOA4 levels were lower in the ACTL group than in the control group (7). However, we could not find data indicating direct interactions between clusterin and APOA2 or APOA4.

In addition to clusterin, several proteins have been identified to non-covalently bind to fibrin clots in the plasma, including haptoglobin (HB), α2-macroglobulin (A2M), and α1-antitrypsin (SERPINA1), which can also act as extracellular chaperones and possibly play a regulatory role in thrombosis and haemostasis by inhibiting fibrin polymerisation and promoting the clearance of unfolded fibrin(ogen) (3). HB exerts anti-inflammatory effects by blocking the inappropriate self-assembly of misfolded extracellular proteins in most body fluids, similar to clusterin (8). Haptoglobin protects various proteins from stress-induced precipitation, and its effects suggest that this activity is relevant *in vivo*. Haptoglobin has been implicated as a biomarker candidate for OA as its serum levels are markedly increased in patients with OA (9).

SERPINA1, α1-microglobulin, clusterin, and HB have been identified as biomarkers of preeclampsia-related serum proteins (10). A2M, which forms stable complexes with misfolded proteins to inhibit their aggregation and precipitation but is unable to independently affect their refolding, can bind to cytokines and growth factors, as well as to the Aβ peptide, prion protein, and β2-microglobulin (B2M), which are associated with diseases such as AD or various forms of amyloidosis (11). A2M acts as an endogenous inhibitor of ADAMTS-7 and ADAMTS-12, enzymes involved in ECM degradation in OA (12). miR-146b plays a critical role in the progression of injury-induced OA by directly targeting A2M (13). These extracellular chaperones might also play a role in controlling amyloid formation and toxicity (11). SERPINA1 and SERPINA3 are implicated as markers of chondrogenic differentiation (14), and it has recently been shown that MMP-3, one of the most important cartilage-degrading collagenase, inactivates α1-antitrypsin, which, owing to its potent chondrogenic and cartilage-protecting capabilities, may have major consequences for OA progression (15, 16). B2M, a component of the major histocompatibility complex type 1, can also contribute to amyloid formation and may affect the musculoskeletal system (17). B2M levels are significantly higher in OA cartilage and synovial fluid than in healthy individuals, suggesting that it could contribute to OA pathogenesis (18). In addition, given the predominant deposition of B2M fibrils on the articular cartilage in OA, serum B2M levels are higher in patients with OA than in healthy controls (19). Therefore, HB, A2M, and B2M may be novel therapeutic targets in patients with OA.

As previously discussed, there is a known interaction between the complement system and clusterin. Complement C3, which plays a role in the activation of the complement system, was found to be highly expressed in OA osteochondral biopsy samples, especially after treatment with IL-1β, and high levels of C3a activation fragments were detected in cartilage and synovium samples (20). Complement factor H (CFH) is a soluble complement inhibitor; several ECM components, such as chondroadherin (21) and the G3 C-type lectin domain of aggrecan (22) are known to interact with CFH. These data indicate that C factors are locally produced and activated in all major joint tissues (cartilage, bone, and synovium) in OA. However, no direct association between C3 and clusterin was found. Lower levels of CD59 (protectin), a potent inhibitor of the complement membrane attack complex (MAC), have been observed in RA and other types of arthritis (23, 24). The complement receptor CR1 has been identified in the synovial fluid of patients with joint inflammation, and probably originates from infiltrating leukocytes (25). CR1 is one of the differentially expressed genes identified between RA and OA patients (26), suggesting that it may be useful as a differential biomarker between different forms of arthritis.

Ceruloplasmin (CP) is a major copper-carrying protein in blood and plays a role in iron metabolism. In the plasma of patients with primary OA, the copper and CP levels were significantly lower than those in patients with RA (27). CP is one of the proteins in stressed serum that is co-purified with clusterin in higher abundance compared to control plasma (1).

Haemopexin (HPX) is an abundant plasma protein involved in the acute-phase response and plays important roles in inflammation and tissue repair by suppressing the release of pro-inflammatory cytokines from macrophages (28). In a study aimed at identifying potential prognostic markers for knee OA using an *N*-glycoproteomic approach, increased levels of HPX glycosylation were observed in patients undergoing disease progression, in a manner similar to clusterin (29). It is plausible to assume that the increased level of glycosylation of HPX and clusterin reduces their anti-inflammatory effects and facilitates disease progression.

Vitamin D-binding protein-macrophage activating factor (GC), present in the blood plasma and other bodily fluids, was identified in the clusterin connectome with a high (26) node degree. This may be due to its ability to bind chondroitin sulphate, a major constituent of cartilage ECM, resulting in multimolecular complexes (30). However, we did not find any data confirming a direct interaction between GC and clusterin.

α-2-HS-glycoprotein (AHSG) is an acute-phase protein which regulates inflammatory responses. In patients with OA, serum AHSG levels were negatively correlated with clinical severity, and lower serum AHSG levels were correlated with a more severe clinical presentation of OA (31). In contrast, higher levels of AHSG were detected in cartilage samples from patients with osteonecrosis of the femoral head, probably to protect the cartilage from the harmful effects of inflammation (32). However, we found no data confirming a direct interaction between AHSG and clusterin.

Cystatin C (CYST3) is a natural endogenous cysteine protease inhibitor that blocks the activity of cathepsins, such as cathepsin K (CatK), and plays a role in the progression of OA. Cystatin C plays a role in neurological disorders involving amyloid deposition, such as AD, and elevated levels of cystatin C have been found in the cerebrospinal fluid of patients with chronic OA (33), although overexpression of cystatin C in OA synovium did not alleviate cartilage pathology in pre-existing OA (34). However, we did not find any data confirming the direct interaction between cystatin C and clusterin.

Vitronectin (VTN, also known as "S protein"), a structural glycoprotein in cartilage ECM, is also present in blood plasma, and it binds to the complement membrane attack complex that has failed to insert into membranes to form the soluble terminal complement complex SC5b-9, which is cleared by the kidneys (35). Clusterin inhibits the assembly of membrane attack complex (36). Similar to clusterin, vitronectin also has extracellular chaperone activity, as it inhibits both Aβ amyloid formation and the amorphous aggregation of citrate synthase (36). V65, a proteolytic fragment of vitronectin, has been implicated as a biomarker of early-stage OA (37). Glycosylated vitronectin, an attractive treatment option for OA, has been reported as a potential candidate for CatK inhibition (38).

Retinol binding protein 4 (RBP4), a member of the lipocalin family and a vitamin A carrier in the blood, has been shown to be produced in OA joints and is associated with higher levels of adipokines and MMPs, indicating that it could be a possible target for disease-modifying OA drugs (DMOADs) (39). The association between obesity indices, plasma RBP4 levels, and clusterin levels was confirmed (40).

Gelsolin (GSN), a protein involved in actin filament assembly and apoptosis regulation, plays a role in amyloid binding/transport processes and has a circulating or plasma isoform, which has been used as a biomarker alongside clusterin, under various conditions, including cancer (41-43), neuropathic pain (44), AD (45), and multiple sclerosis (46). Gelsolin levels were lower in the plasma (47) but higher in the synovial fluid of patients with RA than in non-RA samples (48). The exogenous application of gelsolin, hemopexin, and α1-antitrypsin (acute-phase proteins released by the liver in case of systemic stress) abrogated the effects of IL-1β in an *in vivo* OA model, and intra-articular injection of gelsolin exerted chondroprotective effects in mice with inflammatory arthritis (49). Gelsolin is more abundant in human OA synovial fluid than in RA samples (50), indicating its potential use as a disease biomarker.

Transferrin (TF) is a plasma glycoprotein that mediates iron transport and is one of the most abundant proteins in OA synovial fluid samples (51). However, we did not find any data confirming the direct interaction between transferrin and clusterin.

α1-B glycoprotein (A1BG) is a plasma glycoprotein whose levels have been monitored in various diseases, such as Crohn's disease (52), and it has been proposed as a potential new autoantigen of diagnostic importance for RA (48). α2-glycoprotein 1, zinc-binding (AZGP1) stimulates lipid degradation in adipocytes and is implicated as a prognostic marker for lung cancer (53). Moreover, AZGP1 has been identified in the functional network of genes involved in the pathogenesis of osteochondrosis (54). However, we found no direct links between A1BG, AZGP1, and clusterin in OA.

Leucine-rich α-2-glycoprotein 1 (LRG1) is a new regulator of pathogenic angiogenesis and a novel oncogene-associated protein that contributes to angiogenesis-coupled *de novo* bone formation by promoting angiogenesis and recruiting MSCs to the subchondral bone of OA joints (55). LRG1, along with clusterin, is a candidate biomarker for the diagnosis of various diseases, including acute lymphoblastic leukaemia (56) and ovarian cancer (57).

Lipocalin 2 (LCN2) is a secreted glycoprotein that belongs to a group of transporters of small lipophilic molecules in the circulation. It is an adipose-derived cytokine involved in a number of functions, such as the induction of apoptosis, transport of fatty acids and iron, modulation of inflammation, and metabolic homeostasis. LCN2 has emerged as a useful biomarker of rheumatic diseases (58). As an acute-phase protein, LCN2 is highly relevant as a potential clinical biomarker for low-level systemic inflammatory diseases. LCN2 expression in chondrocytes is modulated by IL-1β, leptin, adiponectin, lipopolysaccharide (LPS), and dexamethasone (59). The synovial fluid levels of LCN2 are significantly higher in patients with RA than in those with OA (60). However, we did not find any data confirming the direct interaction between lipocalin 2 and clusterin.

Tissue inhibitor of metalloproteinase 1 (TIMP1), an inhibitor of matrix metalloproteinases (MMPs) and disintegrin-metalloproteinases (ADAMs and ADAMTSs), is associated with synovial inflammation, radiographic OA severity, and/or OA symptoms (61). TIMP1 is one of the top genes most closely related to OA based on bioinformatics analysis (62). However, we did not find any data confirming a direct interaction between TIMP1 and clusterin.

Secreted phosphoprotein 1 (SPP1; also known as osteopontin) is a sulphated phosphoprotein with cell- and matrix-binding properties that has been identified in human OA chondrocytes (63). Clusterin and osteopontin levels are often monitored together under various conditions, such as asthma (64) and diabetes mellitus (65).

Box-dependent myc-interacting protein 1/bridging integrator 1 (BIN1) and hepatitis A virus cellular receptor 1 (HAVCR1/KIM1) are often used together with clusterin as biomarkers for various conditions (66, 67), but these proteins have not yet been characterised in cartilage and/or OA.

The prevalence of OA is significantly higher among women, which in part may stem from the fact that androgens are believed to exert anti-inflammatory effects in various inflammatory models and in patients with inflammatory diseases (68). Androgen receptor (AR) expression in synovial tissue is correlated with other steroid hormone receptors in OA but not in RA patients (69). Oestrogen receptor β (ERβ) is known to regulate AR signalling, and clusterin is

significantly upregulated in ERβ$^{-/-}$ mice (70), suggesting that the interplay between these pathways may modulate anti-inflammatory pathways through clusterin.

Trefoil factor 3 (TFF3) is a protease-resistant protein with multiple functions, including the inhibition of apoptosis (71). TTF3 is absent from healthy cartilage but is present in OA and induces MMP1, 3, and 13 (72), suggesting its involvement in disease progression. TTF3 is also present in the synovial membrane and synovial fluid of OA patients (73). Although we did not find evidence of direct interactions between TTF3 and clusterin, these two proteins are often monitored together as biomarkers in urine (74, 75).

ABCA7 (ATP-binding cassette, sub-family A (ABC1), member 7), PICALM (phosphatidylinositol-binding clathrin assembly protein), and clusterin have been identified in genome-wide association studies (GWAS) as loci responsible for ~50% of late-onset AD (76, 77).

Ficolin 3 (FCN3, also known as collagen/fibrinogen domain-containing lectin 3, or H-ficolin) is a serum β-2-macroglycoprotein that can activate the complement system *via* the lectin pathway, and its synovial fluid levels in patients with RA are significantly higher than those in patients with OA (78). FCN3, along with clusterin and gelsolin, has been studied as a biomarker for amyotrophic lateral sclerosis (ALS), and serum FCN3 levels have been found to be higher in patients with ALS than in healthy individuals (79).

Vitamin D-dependent calcium-binding protein calbindin 1 (CALB1) is expressed in prechondroblasts, chondroblasts, and newly differentiated chondrocytes (80). Exogenous vitamin D_3 administration upregulates CALB1 expression in renal extracts of mice (81). Although CALB1 is often monitored together with clusterin as a biomarker for various conditions (75), we could not find direct interactions between these two proteins in OA.

We identified three heat shock β proteins (HSPB1, 2, and 3) in the connectome of clusterin. HSPB1 protein expression has been reported to be attenuated by valdecoxib, a non-steroidal anti-inflammatory drug that has been widely used for the treatment of RA and OA, thereby alleviating ER stress (82). However, we found no evidence of a direct association between clusterin and these heat-shock proteins.

BCL-2 is a key regulator of apoptosis and plays a role in OA development (83, 84). Clusterin interacts with Bcl-2 family members, which may represent the molecular basis of its apoptosis-regulatory function (85).

The clusterin connectome in OA using the Cytoscape text-mining approach

We also aimed at identifying interacting partners with clusterin in OA; to this end, we once again employed the text mining service in CytoScape using the keywords ‘clusterin’ and ‘osteoarthritis’. The search returned only 13 interacting partners, of which five entities (AFM, APOA4, HPX, CLU, and C7) were first-shell interactors, and seven proteins (PRG4, MMP3, ORM2, PROC, IGLL5, IGFALS, and SERPINA4) were second-shell interactors (*Table S1*). The first shell interactors were discussed above.

Entity	Protein name	Node degree
First shell interactors		
AFM	alpha-albumin	6
APOA4	apolipoprotein A-4	6
HPX	beta-1B-glycoprotein/hemopexin	5
CLU	clusterin	4
C7	complement component C7	2
Second shell interactors		
PRG4	lubricin	15
MMP3	matrix metallopeptidase 3	11
ORM2	alpha-1-acid glycoprotein 2/oromucosid 2	4
PROC	protein C	3
IGLL5	immunoglobulin lambda like polypeptide 5	1
IGFALS	insulin-like growth factor-binding protein complex acid labile subunit	1
SERPINA4	serpin peptidase inhibitor, clade A, member 4	1

Table S1. Entities in the OA-related connectome network of clusterin ranked by node degree as identified by text-mining using Cytoscape. First and second shell interactors are listed separately.

Among the second-shell interactors, lubricin/proteoglycan 4 (PRG4) has the highest node degree (15). When human OA chondrocytes were cultured in serum-free differentiation media supplemented with TGFβ3, the superficial zone of neocartilage formed contained lubricin and clusterin (86). The plasma levels of clusterin and lubricin peptides can be used as predictive biomarkers for OA (87). Elevated lubricin levels in the synovial fluid may be a potential biomarker for early joint injury prior to the radiographic manifestations of OA (88). Simultaneously, serum lubricin levels significantly decreased after intense exercise in horses (89).

Matrix metallopeptidase 3 (MMP3), a protease involved in the breakdown of cartilage ECM, is detectable in the secretome of pathophysiologically relevant *in vitro* models of OA, together with clusterin (90, 91).

Orosomucoid 2 (ORM2), an acute-phase plasma protein, has recently been identified as one of the six predictive serum biomarkers of response to chondroitin sulfate/glucosamine hydrochloride treatment in patients with knee OA. In fact, ORM2 serum levels at baseline, combined with other relevant variables, could predict patient response to chondroitin sulfate/glucosamine hydrochloride treatment with very high specificity and sensitivity (92).

Protein C (PROC) is a serine protease involved in the degradation of activated forms of coagulation factors V and VIII. Elevated PROC levels are detectable in the synovial fluid of patients with OA and PROC induces aggrecan and collagen proteolysis and release *via* increased MMP activity (93, 94). PROC has been identified as one of the secreted proteins in synovial fluid samples 12 months after anterior cruciate ligament transection (ACLT), and its abundance is correlated with cartilage damage (7). Higher PROC levels were observed in the untreated ACTL group than those in the bridge-enhanced ACL repair group. In contrast, APOA4 levels were lower in the ACTL group. In the same study, increased clusterin and HPX levels were associated with a larger cartilage lesion area.

Immunoglobulin lambda-like polypeptide 5 (IGLL5) is a hub gene associated with the pathogenesis and prognosis of RA (95). However, we did not find any further evidence for an association between IGLL5, clusterin, and OA.

Insulin-like growth factor-1 (IGF-1) is a hormone that regulates skeletal growth and development (96), and increased serum levels are causally related to a higher risk of hip and knee OA (97). Approximately 80% of circulating IGF-1 exists in the form of a complex that plays an important role in regulating the bioavailability of IGF-1 by prolonging its half-life. The complex is composed of IGF-1, IGF-binding protein-3 (IGFBP-3), and insulin-like growth factor-binding protein acid-labile subunit (IGFALS), the latter of which is identified as a second-shell interactor for clusterin in the context of OA. IGFALS has been associated with skeletal defects such as idiopathic short stature (98). SERPINA4 (also known as kallistatin) is a multifunctional protein produced by the liver, and its plasma levels may serve as prognostic or diagnostic biomarkers for various conditions such as chronic liver disease (99) and lung cancer (100). In a study aimed at identifying plasma biomarkers to predict radiographic progression of knee OA, several factors, including clusterin, SERPINA4, and IGFALS levels, were monitored in matched serum and synovial fluid levels using mass spectrometry (87). However, neither SERPINA4 nor IGFALS performed well in this study. Instead, a combined analysis of clusterin and lubricin peptide levels in the plasma has been suggested as a predictive biomarker of OA progression.

References

1. Wyatt AR, Wilson MR. Identification of human plasma proteins as major clients for the extracellular chaperone clusterin. J Biol Chem. 2010;285(6):3532-9.
2. Montanola A, de Retana SF, Lopez-Rueda A, Merino-Zamorano C, Penalba A, Fernandez-Alvarez P, et al. ApoA1, ApoJ and ApoE Plasma Levels and Genotype Frequencies in Cerebral Amyloid Angiopathy. Neuromolecular Med. 2016;18(1):99-108.
3. Talens S, Leebeek FWG, Veerhuis R, Rijken DC. Decoration of Fibrin with Extracellular Chaperones. Thromb Haemost. 2019;119(10):1624-31.
4. Yanagisawa A, Ueda M, Sueyoshi T, Nakamura E, Tasaki M, Suenaga G, et al. Knee osteoarthritis associated with different kinds of amyloid deposits and the impact of aging on type of amyloid. Amyloid. 2016;23(1):26-32.
5. Ayub A, Mackness MI, Arrol S, Mackness B, Patel J, Durrington PN. Serum paraoxonase after myocardial infarction. Arterioscler Thromb Vasc Biol. 1999;19(2):330-5.
6. Barreto G, Soliymani R, Baumann M, Waris E, Eklund KK, Zenobi-Wong M, et al. Functional analysis of synovial fluid from osteoarthritic knee and carpometacarpal joints unravels different molecular profiles. Rheumatology (Oxford). 2019;58(5):897-907.
7. Kiapour AM, Sieker JT, Proffen BL, Lam TT, Fleming BC, Murray MM. Synovial fluid proteome changes in ACL injury-induced posttraumatic osteoarthritis: Proteomics analysis of porcine knee synovial fluid. PLoS One. 2019;14(3):e0212662.
8. Yerbury JJ, Rybchyn MS, Easterbrook-Smith SB, Henriques C, Wilson MR. The acute phase protein haptoglobin is a mammalian extracellular chaperone with an action similar to clusterin. Biochemistry. 2005;44(32):10914-25.

9. Fernandez-Puente P, Calamia V, Gonzalez-Rodriguez L, Lourido L, Camacho-Encina M, Oreiro N, et al. Multiplexed mass spectrometry monitoring of biomarker candidates for osteoarthritis. J Proteomics. 2017;152:216-25.
10. Hsu TY, Hsieh TT, Yang KD, Tsai CC, Ou CY, Cheng BH, et al. Proteomic profiling reveals alpha1-antitrypsin, alpha1-microglobulin, and clusterin as preeclampsia-related serum proteins in pregnant women. Taiwan J Obstet Gynecol. 2015;54(5):499-504.
11. Wilson MR, Yerbury JJ, Poon S. Potential roles of abundant extracellular chaperones in the control of amyloid formation and toxicity. Mol Biosyst. 2008;4(1):42-52.
12. Luan Y, Kong L, Howell DR, Ilalov K, Fajardo M, Bai XH, et al. Inhibition of ADAMTS-7 and ADAMTS-12 degradation of cartilage oligomeric matrix protein by alpha-2-macroglobulin. Osteoarthritis Cartilage. 2008;16(11):1413-20.
13. Liu X, Liu L, Zhang H, Shao Y, Chen Z, Feng X, et al. MiR-146b accelerates osteoarthritis progression by targeting alpha-2-macroglobulin. Aging (Albany NY). 2019;11(16):6014-28.
14. Boeuf S, Steck E, Pelttari K, Hennig T, Buneb A, Benz K, et al. Subtractive gene expression profiling of articular cartilage and mesenchymal stem cells: serpins as cartilage-relevant differentiation markers. Osteoarthritis Cartilage. 2008;16(1):48-60.
15. Kaneva MK. Neutrophil elastase and its inhibitors-overlooked players in osteoarthritis. FEBS J. 2022;289(1):113-6.
16. Wilkinson DJ, Falconer AMD, Wright HL, Lin H, Yamamoto K, Cheung K, et al. Matrix metalloproteinase-13 is fully activated by neutrophil elastase and inactivates its serpin inhibitor, alpha-1 antitrypsin: Implications for osteoarthritis. FEBS J. 2022;289(1):121-39.
17. Wininger AE, Phelps BM, Le JT, Harris JD, Trachtenberg BH, Liberman SR. Musculoskeletal pathology as an early warning sign of systemic amyloidosis: a systematic review of amyloid deposition and orthopedic surgery. BMC Musculoskelet Disord. 2021;22(1):51.
18. Zhang H, Liew CC, Marshall KW. Microarray analysis reveals the involvement of beta-2 microglobulin (B2M) in human osteoarthritis. Osteoarthritis Cartilage. 2002;10(12):950-60.
19. Melikoglu MA, Yildirim K, Senel K. Relationship between radiographic grading of osteoarthritis and serum beta-2 microglobulin. Ir J Med Sci. 2009;178(2):151-4.
20. Assirelli E, Pulsatelli L, Dolzani P, Mariani E, Lisignoli G, Addimanda O, et al. Complement Expression and Activation in Osteoarthritis Joint Compartments. Front Immunol. 2020;11:535010.
21. Sjoberg AP, Manderson GA, Morgelin M, Day AJ, Heinegard D, Blom AM. Short leucine-rich glycoproteins of the extracellular matrix display diverse patterns of complement interaction and activation. Mol Immunol. 2009;46(5):830-9.
22. Melin Furst C, Morgelin M, Vadstrup K, Heinegard D, Aspberg A, Blom AM. The C-type lectin of the aggrecan G3 domain activates complement. PLoS One. 2013;8(4):e61407.
23. Konttinen YT, Ceponis A, Meri S, Vuorikoski A, Kortekangas P, Sorsa T, et al. Complement in acute and chronic arthritides: assessment of C3c, C9, and protectin (CD59) in synovial membrane. Ann Rheum Dis. 1996;55(12):888-94.
24. Wang Q, Rozelle AL, Lepus CM, Scanzello CR, Song JJ, Larsen DM, et al. Identification of a central role for complement in osteoarthritis. Nat Med. 2011;17(12):1674-9.
25. Sadallah S, Lach E, Lutz HU, Schwarz S, Guerne PA, Schifferli JA. CR1, CD35 in synovial fluid from patients with inflammatory joint diseases. Arthritis Rheum. 1997;40(3):520-6.

26. Li WC, Bai L, Xu Y, Chen H, Ma R, Hou WB, et al. Identification of differentially expressed genes in synovial tissue of rheumatoid arthritis and osteoarthritis in patients. J Cell Biochem. 2019;120(3):4533-44.
27. Milanino R, Frigo A, Bambara LM, Marrella M, Moretti U, Pasqualicchio M, et al. Copper and zinc status in rheumatoid arthritis: studies of plasma, erythrocytes, and urine, and their relationship to disease activity markers and pharmacological treatment. Clin Exp Rheumatol. 1993;11(3):271-81.
28. Liang X, Lin T, Sun G, Beasley-Topliffe L, Cavaillon JM, Warren HS. Hemopexin down-regulates LPS-induced proinflammatory cytokines from macrophages. J Leukoc Biol. 2009;86(2):229-35.
29. Fukuda I, Ishihara T, Ohmachi S, Sakikawa I, Morita A, Ikeda M, et al. Potential plasma biomarkers for progression of knee osteoarthritis using glycoproteomic analysis coupled with a 2D-LC-MALDI system. Proteome Sci. 2012;10(1):36.
30. Ruggiero M, Reinwald H, Pacini S. Is chondroitin sulfate responsible for the biological effects attributed to the GC protein-derived Macrophage Activating Factor (GcMAF)? Med Hypotheses. 2016;94:126-31.
31. Favero M, Belluzzi E, Frallonardo P, Peruzzo L, Tauro L, Oliviero F, et al. Synovial fluid fetuin-A levels in patients affected by osteoarthritis with or without evidence of calcium crystals. Rheumatology (Oxford). 2019;58(4):729-30.
32. Song J, Wu J, Poulet B, Liang J, Bai C, Dang X, et al. Proteomics analysis of hip articular cartilage identifies differentially expressed proteins associated with osteonecrosis of the femoral head. Osteoarthritis Cartilage. 2021;29(7):1081-92.
33. Guo SL, Han CT, Jung JL, Chen WJ, Mei JJ, Lee HC, et al. Cystatin C in cerebrospinal fluid is upregulated in elderly patients with chronic osteoarthritis pain and modulated through matrix metalloproteinase 9-specific pathway. Clin J Pain. 2014;30(4):331-9.
34. Kyostio-Moore S, Piraino S, Berthelette P, Moran N, Serriello J, Bendele A, et al. Overexpression of cystatin C in synovium does not reduce synovitis or cartilage degradation in established osteoarthritis. Arthritis Res Ther. 2015;17:5.
35. Ogawa T, Yorioka N, Yamakido M. Immunohistochemical studies of vitronectin, C5b-9, and vitronectin receptor in membranous nephropathy. Nephron. 1994;68(1):87-96.
36. Geraghty NJ, Satapathy S, Kelly M, Cheng F, Lee A, Wilson MR. Expanding the family of extracellular chaperones: Identification of human plasma proteins with chaperone activity. Protein Sci. 2021;30(11):2272-86.
37. Ourradi K, Xu Y, de Seny D, Kirwan J, Blom A, Sharif M. Development and validation of novel biomarker assays for osteoarthritis. PLoS One. 2017;12(7):e0181334.
38. Date K, Sakagami H, Yura K. Regulatory properties of vitronectin and its glycosylation in collagen fibril formation and collagen-degrading enzyme cathepsin K activity. Sci Rep. 2021;11(1):12023.
39. Scotece M, Koskinen-Kolasa A, Pemmari A, Leppanen T, Hamalainen M, Moilanen T, et al. Novel adipokine associated with OA: retinol binding protein 4 (RBP4) is produced by cartilage and is correlated with MMPs in osteoarthritis patients. Inflamm Res. 2020;69(4):415-21.
40. Won JC, Park CY, Oh SW, Lee ES, Youn BS, Kim MS. Plasma clusterin (ApoJ) levels are associated with adiposity and systemic inflammation. PLoS One. 2014;9(7):e103351.
41. Cohen A, Wang E, Chisholm KA, Kostyleva R, O'Connor-McCourt M, Pinto DM. A mass spectrometry-based plasma protein panel targeting the tumor microenvironment in patients with breast cancer. J Proteomics. 2013;81:135-47.

42. Gajbhiye A, Dabhi R, Taunk K, Vannuruswamy G, RoyChoudhury S, Adhav R, et al. Urinary proteome alterations in HER2 enriched breast cancer revealed by multipronged quantitative proteomics. Proteomics. 2016;16(17):2403-18.
43. Rithidech KN, Honikel L, Simon SR. Radiation leukemogenesis: a proteomic approach. Exp Hematol. 2007;35(4 Suppl 1):117-24.
44. Lind AL, Emami Khoonsari P, Sjodin M, Katila L, Wetterhall M, Gordh T, et al. Spinal Cord Stimulation Alters Protein Levels in the Cerebrospinal Fluid of Neuropathic Pain Patients: A Proteomic Mass Spectrometric Analysis. Neuromodulation. 2016;19(6):549-62.
45. Thambisetty M. Do extracellular chaperone proteins in plasma have potential as Alzheimer's disease biomarkers? Biomark Med. 2010;4(6):831-4.
46. Rithidech KN, Honikel L, Milazzo M, Madigan D, Troxell R, Krupp LB. Protein expression profiles in pediatric multiple sclerosis: potential biomarkers. Mult Scler. 2009;15(4):455-64.
47. Osborn TM, Verdrengh M, Stossel TP, Tarkowski A, Bokarewa M. Decreased levels of the gelsolin plasma isoform in patients with rheumatoid arthritis. Arthritis Res Ther. 2008;10(5):R117.
48. Biswas S, Sharma S, Saroha A, Bhakuni DS, Malhotra R, Zahur M, et al. Identification of novel autoantigen in the synovial fluid of rheumatoid arthritis patients using an immunoproteomics approach. PLoS One. 2013;8(2):e56246.
49. Kaneva MK, Greco KV, Headland SE, Montero-Melendez T, Mori P, Greenslade K, et al. Identification of Novel Chondroprotective Mediators in Resolving Inflammatory Exudates. J Immunol. 2017;198(7):2876-85.
50. Mateos J, Lourido L, Fernandez-Puente P, Calamia V, Fernandez-Lopez C, Oreiro N, et al. Differential protein profiling of synovial fluid from rheumatoid arthritis and osteoarthritis patients using LC-MALDI TOF/TOF. J Proteomics. 2012;75(10):2869-78.
51. Balakrishnan L, Nirujogi RS, Ahmad S, Bhattacharjee M, Manda SS, Renuse S, et al. Proteomic analysis of human osteoarthritis synovial fluid. Clin Proteomics. 2014;11(1):6.
52. Gazouli M, Anagnostopoulos AK, Papadopoulou A, Vaiopoulou A, Papamichael K, Mantzaris G, et al. Serum protein profile of Crohn's disease treated with infliximab. J Crohns Colitis. 2013;7(10):e461-70.
53. Wu X, Wang L, Feng F, Tian S. Weighted gene expression profiles identify diagnostic and prognostic genes for lung adenocarcinoma and squamous cell carcinoma. J Int Med Res. 2020;48(3):300060519893837.
54. Rangkasenee N, Murani E, Schellander K, Cinar MU, Ponsuksili S, Wimmers K. Gene expression profiling of articular cartilage reveals functional pathways and networks of candidate genes for osteochondrosis in pigs. Physiol Genomics. 2013;45(18):856-65.
55. Wang Y, Xu J, Zhang X, Wang C, Huang Y, Dai K, et al. TNF-alpha-induced LRG1 promotes angiogenesis and mesenchymal stem cell migration in the subchondral bone during osteoarthritis. Cell Death Dis. 2017;8(3):e2715.
56. Cavalcante Mde S, Torres-Romero JC, Lobo MD, Moreno FB, Bezerra LP, Lima DS, et al. A panel of glycoproteins as candidate biomarkers for early diagnosis and treatment evaluation of B-cell acute lymphoblastic leukemia. Biomark Res. 2016;4:1.
57. Wu J, Xie X, Nie S, Buckanovich RJ, Lubman DM. Altered expression of sialylated glycoproteins in ovarian cancer sera using lectin-based ELISA assay and quantitative glycoproteomics analysis. J Proteome Res. 2013;12(7):3342-52.

58. Abella V, Scotece M, Conde J, Gomez R, Lois A, Pino J, et al. The potential of lipocalin-2/NGAL as biomarker for inflammatory and metabolic diseases. Biomarkers. 2015;20(8):565-71.
59. Conde J, Gomez R, Bianco G, Scotece M, Lear P, Dieguez C, et al. Expanding the adipokine network in cartilage: identification and regulation of novel factors in human and murine chondrocytes. Ann Rheum Dis. 2011;70(3):551-9.
60. Katano M, Okamoto K, Arito M, Kawakami Y, Kurokawa MS, Suematsu N, et al. Implication of granulocyte-macrophage colony-stimulating factor induced neutrophil gelatinase-associated lipocalin in pathogenesis of rheumatoid arthritis revealed by proteome analysis. Arthritis Res Ther. 2009;11(1):R3.
61. Haraden CA, Huebner JL, Hsueh MF, Li YJ, Kraus VB. Synovial fluid biomarkers associated with osteoarthritis severity reflect macrophage and neutrophil related inflammation. Arthritis Res Ther. 2019;21(1):146.
62. Zhu Z, Zhong L, Li R, Liu Y, Chen X, Li Z, et al. Study of Osteoarthritis-Related Hub Genes Based on Bioinformatics Analysis. Biomed Res Int. 2020;2020:2379280.
63. Pullig O, Weseloh G, Gauer S, Swoboda B. Osteopontin is expressed by adult human osteoarthritic chondrocytes: protein and mRNA analysis of normal and osteoarthritic cartilage. Matrix Biol. 2000;19(3):245-55.
64. Dombai B, Ivancso I, Bikov A, Oroszi D, Bohacs A, Muller V, et al. Circulating Clusterin and Osteopontin Levels in Asthma and Asthmatic Pregnancy. Can Respir J. 2017;2017:1602039.
65. Maser RE, James Lenhard M, Pohlig RT, Babu Balagopal P. Osteopontin and clusterin levels in type 2 diabetes mellitus: differential association with peripheral autonomic nerve function. Neurol Sci. 2017;38(9):1645-50.
66. Almeida JFF, Dos Santos LR, Trancozo M, de Paula F. Updated Meta-Analysis of BIN1, CR1, MS4A6A, CLU, and ABCA7 Variants in Alzheimer's Disease. J Mol Neurosci. 2018;64(3):471-7.
67. Vinken P, Starckx S, Barale-Thomas E, Looszova A, Sonee M, Goeminne N, et al. Tissue Kim-1 and urinary clusterin as early indicators of cisplatin-induced acute kidney injury in rats. Toxicol Pathol. 2012;40(7):1049-62.
68. Montano LM, Flores-Soto E, Sommer B, Solis-Chagoyan H, Perusquia M. Androgens are effective bronchodilators with anti-inflammatory properties: A potential alternative for asthma therapy. Steroids. 2020;153:108509.
69. Capellino S, Riepl B, Rauch L, Angele P, Cutolo M, Straub RH. Quantitative determination of steroid hormone receptor positive cells in the synovium of patients with rheumatoid arthritis and osteoarthritis: is there a link to inflammation? Ann Rheum Dis. 2007;66(1):53-8.
70. Wu WF, Maneix L, Insunza J, Nalvarte I, Antonson P, Kere J, et al. Estrogen receptor beta, a regulator of androgen receptor signaling in the mouse ventral prostate. Proc Natl Acad Sci U S A. 2017;114(19):E3816-E22.
71. Taupin DR, Kinoshita K, Podolsky DK. Intestinal trefoil factor confers colonic epithelial resistance to apoptosis. Proc Natl Acad Sci U S A. 2000;97(2):799-804.
72. Rosler S, Haase T, Claassen H, Schulze U, Schicht M, Riemann D, et al. Trefoil factor 3 is induced during degenerative and inflammatory joint disease, activates matrix metalloproteinases, and enhances apoptosis of articular cartilage chondrocytes. Arthritis Rheum. 2010;62(3):815-25.
73. Popp J, Schicht M, Garreis F, Klinger P, Gelse K, Sesselmann S, et al. Human Synovia Contains Trefoil Factor Family (TFF) Peptides 1-3 Although Synovial Membrane Only Produces TFF3: Implications in Osteoarthritis and Rheumatoid Arthritis. Int J Mol Sci. 2019;20(23).

74. Griffin BR, Faubel S, Edelstein CL. Biomarkers of Drug-Induced Kidney Toxicity. Ther Drug Monit. 2019;41(2):213-26.
75. Wu CY, Yang HY, Chien HP, Tseng MH, Huang JL. Urinary clusterin-a novel urinary biomarker associated with pediatric lupus renal histopathologic features and renal survival. Pediatr Nephrol. 2018;33(7):1189-98.
76. Darawi MN, Ai-Vyrn C, Ramasamy K, Hua PP, Pin TM, Kamaruzzaman SB, et al. Allele-specific polymerase chain reaction for the detection of Alzheimer's disease-related single nucleotide polymorphisms. BMC Med Genet. 2013;14:27.
77. Morgan K. The three new pathways leading to Alzheimer's disease. Neuropathol Appl Neurobiol. 2011;37(4):353-7.
78. Ammitzboll CG, Thiel S, Ellingsen T, Deleuran B, Jorgensen A, Jensenius JC, et al. Levels of lectin pathway proteins in plasma and synovial fluid of rheumatoid arthritis and osteoarthritis. Rheumatol Int. 2012;32(5):1457-63.
79. Mohanty L, Henderson RD, McCombe PA, Lee A. Levels of clusterin, CD5L, ficolin-3, and gelsolin in ALS patients and controls. Amyotroph Lateral Scler Frontotemporal Degener. 2020;21(7-8):631-4.
80. Balmain N, von Eichel B, Toury R, Belquasmi F, Hauchecorne M, Klaus G, et al. Calbindin-D28K and -D9K and 1,25(OH)2 vitamin D3 receptor immunolocalization and mineralization induction in long-term primary cultures of rat epiphyseal chondrocytes. Bone. 1995;17(1):37-45.
81. Xue Y, Karaplis AC, Hendy GN, Goltzman D, Miao D. Exogenous 1,25-dihydroxyvitamin D3 exerts a skeletal anabolic effect and improves mineral ion homeostasis in mice that are homozygous for both the 1alpha-hydroxylase and parathyroid hormone null alleles. Endocrinology. 2006;147(10):4801-10.
82. Kim TJ, Lee HJ, Pyun DH, Abd El-Aty AM, Jeong JH, Jung TW. Valdecoxib improves lipid-induced skeletal muscle insulin resistance via simultaneous suppression of inflammation and endoplasmic reticulum stress. Biochem Pharmacol. 2021;188:114557.
83. Caric D, Zekic Tomas S, Filipovic N, Soljic V, Benzon B, Glumac S, et al. Expression Pattern of iNOS, BCL-2 and MMP-9 in the Hip Synovium Tissue of Patients with Osteoarthritis. Int J Mol Sci. 2021;22(3).
84. Li X, Fu X, Gao Y, Li H, Wang W, Shen Y. Expression of tissue inhibitor of metalloproteinases-1 and B-cell lymphoma-2 in the synovial membrane in patients with knee osteoarthritis. Exp Ther Med. 2018;15(1):885-9.
85. Lee DH, Ha JH, Kim Y, Bae KH, Park JY, Choi WS, et al. Interaction of a putative BH3 domain of clusterin with anti-apoptotic Bcl-2 family proteins as revealed by NMR spectroscopy. Biochem Biophys Res Commun. 2011;408(4):541-7.
86. Bianchi VJ, Weber JF, Waldman SD, Backstein D, Kandel RA. Formation of Hyaline Cartilage Tissue by Passaged Human Osteoarthritic Chondrocytes. Tissue Eng Part A. 2017;23(3-4):156-65.
87. Ritter SY, Collins J, Krastins B, Sarracino D, Lopez M, Losina E, et al. Mass spectrometry assays of plasma biomarkers to predict radiographic progression of knee osteoarthritis. Arthritis Res Ther. 2014;16(5):456.
88. Wang Y, Gludish DW, Hayashi K, Todhunter RJ, Krotscheck U, Johnson PJ, et al. Synovial fluid lubricin increases in spontaneous canine cruciate ligament rupture. Sci Rep. 2020;10(1):16725.
89. Matheson A, Regmi SC, Jay GD, Schmidt TA, Scott WM. The Effect of Intense Exercise on Equine Serum Proteoglycan-4/Lubricin. Front Vet Sci. 2020;7:599287.

90. Clutterbuck AL, Smith JR, Allaway D, Harris P, Liddell S, Mobasheri A. High throughput proteomic analysis of the secretome in an explant model of articular cartilage inflammation. J Proteomics. 2011;74(5):704-15.
91. Matta C, Fellows CR, Quasnichka H, Williams A, Jeremiasse B, Allaway D, et al. Clusterin secretion is attenuated by the proinflammatory cytokines interleukin-1beta and tumor necrosis factor-alpha in models of cartilage degradation. J Orthop Res. 2021;39(5):1017-29.
92. Blanco FJ, Camacho-Encina M, Gonzalez-Rodriguez L, Rego-Perez I, Mateos J, Fernandez-Puente P, et al. Predictive modeling of therapeutic response to chondroitin sulfate/glucosamine hydrochloride in knee osteoarthritis. Ther Adv Chronic Dis. 2019;10:2040622319870013.
93. Jackson MT, Moradi B, Smith MM, Jackson CJ, Little CB. Activation of matrix metalloproteinases 2, 9, and 13 by activated protein C in human osteoarthritic cartilage chondrocytes. Arthritis Rheumatol. 2014;66(6):1525-36.
94. Jackson MT, Smith MM, Smith SM, Jackson CJ, Xue M, Little CB. Activation of cartilage matrix metalloproteinases by activated protein C. Arthritis Rheum. 2009;60(3):780-91.
95. Wu B, He Y, Yang D, Liu RX. Identification of hub genes and therapeutic drugs in rheumatoid arthritis patients. Clin Rheumatol. 2021;40(8):3299-309.
96. Laron Z. Insulin-like growth factor 1 (IGF-1): a growth hormone. Mol Pathol. 2001;54(5):311-6.
97. Hartley A, Sanderson E, Paternoster L, Teumer A, Kaplan RC, Tobias JH, et al. Mendelian randomization provides evidence for a causal effect of higher serum IGF-1 concentration on risk of hip and knee osteoarthritis. Rheumatology (Oxford). 2021;60(4):1676-86.
98. Kang MJ. Novel genetic cause of idiopathic short stature. Ann Pediatr Endocrinol Metab. 2017;22(3):153-7.
99. Shashidhar KN, Lakshmaiah V, Muninarayana C, Nallagangula KS. Quantitative ELISA for SERPINA4/kallistatin. Biotechniques. 2021;71(5):556-65.
100. Kim YI, Ahn JM, Sung HJ, Na SS, Hwang J, Kim Y, et al. Meta-markers for the differential diagnosis of lung cancer and lung disease. J Proteomics. 2016;148:36-43.
